# A scoping review of human digital twins in healthcare applications and usage patterns

**DOI:** 10.1038/s41746-025-01910-w

**Published:** 2025-09-30

**Authors:** Brant H. Tudor, Ryan Shargo, Geoffrey M. Gray, Jamie L. Fierstein, Frederick H. Kuo, Robert Burton, Joyce T. Johnson, Brandi B. Scully, Alfred Asante-Korang, Mohamed A. Rehman, Luis M. Ahumada

**Affiliations:** 1 Center for Pediatric Data Science and Analytic Methodology, Johns Hopkins All Children’s Hospital, St. Petersburg, FL USA; 2 Department of Anesthesiology and Critical Care Medicine, Johns Hopkins University School of Medicine, Baltimore, MD USA; 3 University of South Florida, Morsani College of Medicine, Tampa, FL USA; 4 Department of Anesthesia and Pain Medicine, Johns Hopkins All Children’s Hospital, St. Petersburg, FL USA; 5 Epidemiology and Biostatistics Shared Resource, Institute for Clinical and Translational Research, Johns Hopkins All Children’s Hospital, St. Petersburg, FL USA; 6 Clark University, Worcester, MA USA; 7 Heart Institute, Johns Hopkins All Children’s Hospital, St. Petersburg, FL USA; 8 Division of Pediatric Cardiac Surgery, Department of Cardiac Surgery, Johns Hopkins University School of Medicine, Baltimore, MD USA

**Keywords:** Disease prevention, Lifestyle modification, Preventive medicine, Biomedical engineering, Mathematics and computing, Computational science, Computer science, Information technology, Software

## Abstract

Digital twins have become increasingly popular across various industries as dynamic virtual models of physical systems. In healthcare, Human Digital Twins (HDTs) serve as virtual counterparts to patients. According to the National Academies of Sciences, Engineering, and Medicine (NASEM), a digital twin must be personalized, dynamically updated, and have predictive capabilities to—in the context of health care—inform clinical decision-making. This scoping review aims to assess the current state of HDTs in healthcare, examining whether the literature aligns with the NASEM definition and identifying trends. A systematic literature search was conducted, covering articles published from January 2017 to July 2024. Only 18 of the 149 included studies (12.08%) fully met the NASEM digital twin criteria. Digital shadows made up 9.4% of studies, general digital models comprised 10.07%, and virtual patient cohorts were another 10.07%. Only two studies mentioned verification, validation, and uncertainty quantification (VVUQ), a critical NASEM standard for model reliability.

## Introduction

Digital twins have emerged as a powerful technology for constructing virtual representations of physical assets, processes, and systems. Digital twins were first described by Dr. Michael Grieves in a 2002 engineering presentation at the University of Michigan regarding Product Lifecycle Management (PLM)^1^. The framework consisted of three elements: a physical system, a virtual representation of the physical system, and the link for bidirectional information flow between the two. The virtual representation of the physical system serves as a mirror or “twin” of the physical, being dynamically updated by the physical system and vice versa^1^. The term “digital twin” itself was later coined in 2010 by NASA in the context of aerospace engineering through an initiative for a fundamental paradigm shift in the management of air force vehicles^2^.

Since then, digital twins have been widely adopted in various fields of engineering to enable instantaneous forecasting of system behaviors, allowing engineers to anticipate and respond to potential issues before they arise. These virtual models facilitate performance enhancement by identifying optimal operating parameters and processes as well as dynamically tracking the health of their physical counterparts, providing updates as needed. For example, a digital twin of a missile may model the missile in real-time using sensors on the physical object to predict aerial trajectory and provide automatic adjustments to the missile’s flight path. The application of digital twins has expanded in recent years with the development and synergy of technologies including Generative Artificial Intelligence (AI), Cognitive Computing (CC), Internet of Things (IoT), and advanced sensor systems, allowing for more feasible real-time updates from physical to virtual and vice versa^3^.

Recently, digital twin applications have entered the realm of healthcare. Katsoulakis et al. noted a substantial increase in publications referencing digital twins on PubMed since 2017^3^. As the term has evolved to encompass multiple use cases in a variety of disciplines, the term “digital twin” has shifted away from its aeronautical roots. Other terms such as “digital model” and “digital shadow” have emerged to describe varying degrees of connectivity between the physical twin and its virtual counterpart^4^. Whereas a digital twin entails a dynamic, bidirectional exchange of information, a digital model refers to a digital representation of a physical system with no automatic data exchange, while a digital shadow describes a system with one-way, real-time data flow from the physical to the virtual environment^4^.

In healthcare, Human Digital Twins (HDTs) have realized value in the modeling of human organs, organ systems, and cellular interactions^5^. However, as applications of digital twins have expanded, the term has been applied inconsistently to describe a variety of models ranging from high fidelity, generalized, mechanistic models of the heart^6^ to personalized predictive algorithms for interventions in cancer treatment^7^. In some instances, models labeled as digital twins lack the core, defining features and may be more accurately categorized as digital models or digital shadows. Other times, the label of a digital twin is inappropriately assigned to virtually generated patients or organs utilized for in-silico clinical trials^8^, better described as virtual patient cohorts^9^. Thus, the application of such definitions to a human system presents challenges as authors navigate what it means to dynamically or automatically update a person^5^. Whereas technological applications such as missile trajectory can be automatically updated in real-time, updating a human system without the use of implantable IoT devices poses difficulties, typically necessitating a human-in-the-loop approach to carrying out decisions.

To clarify the definition and standardize usage across disciplines, the National Academies of Sciences, Engineering, and Medicine (NASEM) came together to set a standard definition for digital twins in their 2024 report entitled, “Foundational Research Gaps and Future Directions for Digital Twins.”^10^ Therein, a digital twin is defined:

“A digital twin is a set of virtual information constructs that mimics the structure, context, and behavior of a natural, engineered, or social system (or system-of-systems), is dynamically updated with data from its physical twin, has a predictive capability, and informs decisions that realize value. The bidirectional interaction between the virtual and the physical is central to the digital twin”^10^.

This definition emphasizes the personalization of the digital twin to a specific patient system, allowing for dynamic updates between physical and virtual and the capability to inform decisions that realize value. Additionally, the NASEM emphasizes the importance of verification, validation, and uncertainty quantification (VVUQ) in the development of digital twins. VVUQ has previously been described by the National Research Council (NRC) in the context of computational models to evaluate trustworthiness and quality of model predictions^11^.

Given their rising popularity, this study conducted a scoping review of the literature on digital twins in healthcare, particularly HDTs, to clarify how the term is being used, what systems are being modeled, and for what purposes. We utilized the definition of “digital twin” provided by the NASEM to evaluate whether proposed digital twin models are in accordance with NASEM guidelines. We extended these requirements to HDTs by evaluating whether such twins are personalized to the patient, dynamically updated between physical and virtual systems, and are utilized in clinical decision support. Additionally, we distinguished between human-in-the-loop recommendations and automatic updates to the physical system. This manuscript serves as a review of the current literature regarding HDTs in healthcare and as a summary of current trends in HDT use and model designs. We aimed to investigate whether the term “digital twin” is being used incorrectly or imprecisely within the literature and to assess the consistency and accuracy of its application in various contexts.

## Results

### Study selection

**Fig. 1:**
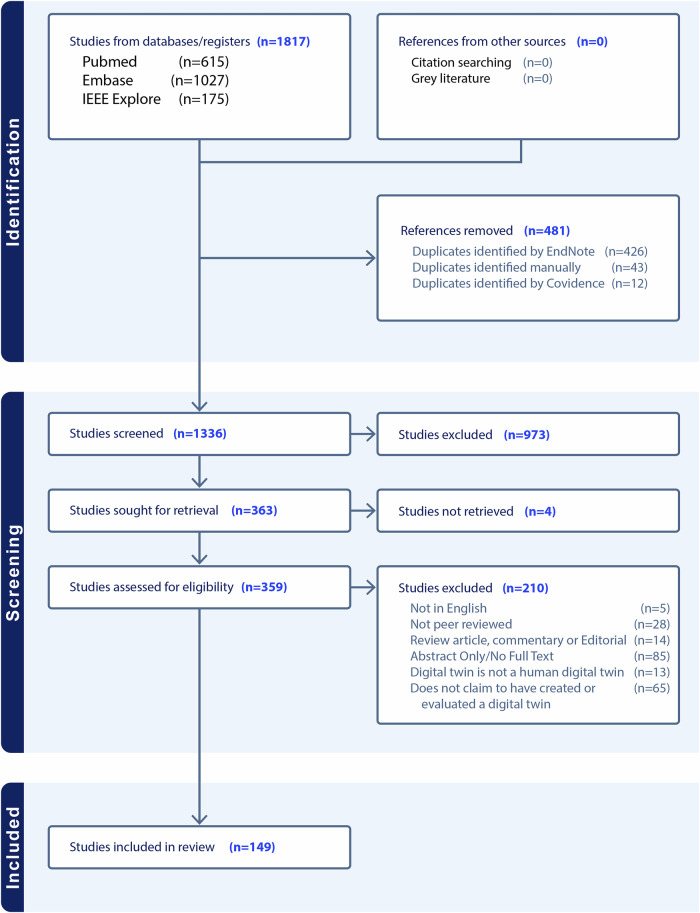
PRISMA flow diagram for article identification, screening, and inclusion. 481 references were excluded because they were duplicates, and 1187 were removed during screening, leaving 149 studies to be evaluated for the purposes of this review.

Overall, we identified 1817 records from database searching. We removed 481 duplicates. Following title and abstract screening, we sought retrieval for 363 articles based on their mention of digital twins in healthcare. We were unable to retrieve four articles. Of the remaining 359 articles, 210 were excluded for the following reasons: 85 lacked full-text availability; 65 did not claim to create or evaluate a HDT; 28 were non-peer-reviewed pre-prints; 14 were review articles, commentaries, or editorials; 13 evaluated non-human digital twins; and five were not published in English. This yielded 149 articles for final inclusion. A full-flow diagram assessing study identification, screening, and inclusion can be seen in Fig. 1.

### Study characteristics

This scoping review included 149 studies published between 2017 and 2024^6–8,12–157^. Of these, the majority of authors were affiliated with academic or medical institutions (*n* = 130, 87.25%), followed by industry (*n* = 14, 9.40%) and government organizations (*n* = 5, 3.35%). Most models studied were empirical in design (*n* = 64, 42.95%), with fewer classified as mechanistic (*n* = 47, 31.55%) or hybrid (*n* = 38, 25.50%). Of the articles that claimed to have created or evaluated digital twins, the most common model type was a personalized digital model that was not dynamically updated or used for decision support (*n* = 56, 37.58%). The second most common type of model was a personalized digital model that was not dynamically updated and was used once for decision support (*n* = 31, 20.80%). Eighteen models fulfilled all three criteria set by the NASEM for a digital twin (*n* = 18, 12.08%). Of these, 17 models (11.41%) were classified as digital twins with human-in-the-loop recommendations, whereas only 1 model (0.67%) fulfilled the criteria of a traditional digital twin with automatic updates to the physical twin. Digital shadows (*n* = 14) accounted for 9.40% of models, while general digital models and virtual patient cohorts each comprised 10.07% (*n* = 15 each).

Among the system classifications identified, cardiac models were most prevalent (*n* = 43, 28.86%), followed by models of human metabolism (*n* = 19, 12.75%), and musculoskeletal models (*n* = 18, 12.08%). Of the 19 metabolic studies, 13 were further classified as diabetic, since they modeled insulin and glucose metabolism in the context of patients with diabetes. Of the 18 musculoskeletal models, 13 were further classified as skeletal models, focusing solely on modeling bone. A detailed breakdown of overall study characteristics is presented in Table 1.

**Table 1:** Study characteristics

Author affiliation	*n* (%)
Academic/Medical	130 (87.25)
Industry	14 (9.40)
Government	5 (3.36)
Design of model	*n* (%)
Empirical	64 (42.95)
Mechanistic	47 (31.55)
Hybrid	38 (25.50)
Type of model	*n* (%)
Personalized digital model, not used for decision support	56 (37.58)
Personalized digital model, used once for decision support	31 (20.81)
Digital twin; dynamically updated with human-in-the-loop recommendations	17 (11.41)
Virtual patient cohort	15 (10.07)
General digital model	15 (10.07)
Digital shadow; dynamically updated and not used for decision support	14 (9.40)
Digital twin; dynamically updated, automatic updates to physical system	1 (0.67)
Systems modeled	*n* (%)
Cardiac	43 (28.86)
Metabolic	19 (12.75)
Musculoskeletal	18 (12.08)
Other	14 (9.40)
Cancer	11 (7.38)
Whole body	10 (6.71)
Respiratory	9 (6.04)
Neurological	6 (4.03)
Hepatic	5 (3.36)
Immune	5 (3.36)
Surgical site	4 (2.68)
Epidermal	3 (2.01)
Reproductive	2 (1.34)

Stratification by model type and system classification revealed that most models meeting the NASEM digital twin criteria were either metabolic (*n* = 8) or categorized as “other” (*n* = 4). The majority of cancer models (*n* = 5) and immune models (*n* = 4) were classified as virtual patient cohorts rather than digital twins. Cardiac models made up the largest category of personalized digital models. Of these, a majority (*n* = 24) were personalized digital models that did not provide decision support. A detailed breakdown of cross tabulations between model type and model system classification can be found in Table 2.

**Table 2:** Crosstab study counts by model type/modeled system

	Body	Cancer	Cardiac	Epidermal	Hepatic	Immune	Metabolic	Musculoskeletal	Neurological	Reproductive	Respiratory	Surgical site	Other
Digital twins	1	1	1	3	0	0	8	0	0	0	0	0	4
Digital shadows	2	1	2	0	1	0	1	3	1	1	1	0	1
Personalized digital model (w CDS)	0	4	11	0	1	1	4	3	1	0	4	0	2
Personalized digital model	7	0	24	0	0	0	2	9	3	0	4	4	3
General digital model	0	0	3	0	3	0	2	3	1	1	0	0	2
Virtual patient cohort	0	5	2	0	0	4	2	0	0	0	0	0	2
Total	10	11	43	3	5	5	19	18	6	2	9	4	14
Empirical	7	6	9	0	1	2	10	5	4	1	4	4	11
Mechanistic	1	3	21	3	2	2	1	9	1	1	2	0	1
Hybrid	2	2	13	0	2	1	8	4	1	0	3	0	2
Consumer grade sensors	5	0	3	0	1	0	14	3	0	0	2	3	4
Clinical grade sensors	7	6	35	0	3	2	4	14	5	1	8	1	5
No sensors	0	5	5	3	2	3	1	3	1	1	0	0	6

To better understand the data sources used to inform such HDTs, we analyzed the types of sensors employed across studies. We compiled data to compare the utilization of clinical-grade versus consumer-grade sensors. Among the studies analyzed, 91 (61.06%) employed clinical-grade sensors, 35 (23.49%) utilized consumer-grade sensors, and 30 (20.13%) did not incorporate sensors at all. Notably, seven studies (4.70%) used both sensor types and were therefore included in the counts for both categories. Studies that did not employ sensors instead relied on alternative data sources, such as electronic health records (EHR), medical notes, or registry databases.

In addition to sensors, we examined the role of imaging data in constructing digital models with approximately half of the studies (*n* = 74, 49.66%) incorporating some form of imaging data as a form of input. Among these, 13 studies (8.72%) employed multiple imaging modalities, while four studies (2.68%) used imaging data but did not specify the source. The remaining studies utilized the following imaging types for model construction: computed tomography (CT; *n* = 23, 15.44%), Magnetic Resonance Imaging (MRI) (*n* = 14, 9.40%), X-rays (*n* = 6, 4.03%), video (*n* = 5, 3.36%), ultrasound (*n* = 3, 2.01%), photos/still images (*n* = 2, 1.34%), computer-generated images (CGI; *n* = 1, 0.67%), confocal scanning laser micrographs (*n* = 1, 0.67%), positron emission tomography (PET; *n* = 1, 0.67%), and optical coherence tomography (OCT; *n* = 1, 0.67%). The distribution of empirical, mechanistic, and hybrid models among studies that used imaging data (43.24%, 32.43%, 24.32%, respectively) closely mirrored that of studies without imaging data (42.67%, 30.67%, 26.67%, respectively). Further crosstab counts are reported in Table 2.

Of the models that met the NASEM standard for digital twins, there were eight metabolic studies^18,19,21,25–29^ and three epidermal studies^14,22,24^, while every other twin-type featured in this group was represented by a single study each: body^17^, cancer^23^, cardiac^12^, physical activity^15^, sepsis^16^, lymphedema^20^, and ICU patient care^13^. Of these, ten (55.55%) used consumer grade sensors, five (27.78%) used clinical grade sensors, and three (16.67%) did not use sensors. Only one NASEM digital twin used imaging data, which came in the form of a video signal. As a final consideration, we tabulated explicit mentions of VVUQ; however, this only occurred in two studies^20,64^.

## Discussion

This scoping review evaluated the use of the term “digital twin” in healthcare, with a particular emphasis on HDTs. A secondary objective was to characterize the types of models labeled as “digital twins” across various organ systems and clinical applications, and to assess the extent to which these models adhered to the definition established by the NASEM. A total of 149 studies published between 2017 and 2024 met the inclusion criteria for this review. The results of the current study highlight a significant increase in research interest in digital twins in healthcare. This is supported by findings by Katsoulakis et al., who reported that publications regarding digital twins in healthcare have increased each year from 2017 to 2023, with a rising proportion of studies exploring such healthcare applications relative to the broader digital twin literature^3^. The expanded scholarship on HDTs coincides with the rise of related technologies such as IoT, AI, and advanced sensor systems, allowing for digital twin systems to become more easily accessible and feasible.

To provide context for the types of human models being developed, we established 13 retrospective system categories. Categories were determined based on the organ system or disease process primarily modeled. Studies evaluating cardiac models were the most common (*n* = 43, 28.86%), followed by metabolic models (*n* = 19, 12.75%), and musculoskeletal models (*n* = 18, 12.18%). Diabetic models accounted for over half of metabolic model publications (*n* = 13). Additionally, models focusing on the skeletal system alone predominated in the musculoskeletal model group (*n* = 13). In contrast, models of the epidermis, reproductive system, and surgical surface anatomy were infrequently represented, reflecting more limited HDT development in these domains.

The predominance of cardiac models underscores the research interest in precision cardiac care, mirroring its status as the number one cause of mortality worldwide^158^. The high clinical demand for cardiovascular disease tools, coupled with the relative feasibility of modeling cardiac perfusion and blood flow, helps account for the prevalence of cardiac models. Additionally, the heart’s function is relatively well-understood, and abundant, high-quality data from technologies like electrocardiograms (ECGs), MRI studies, and wearables make it a good candidate for modeling. The availability of quantifiable outcomes, such as heart rate and ejection fraction, allow for easier validation of models, driving their widespread development and adoption.

Similarly, metabolic digital twin models were likely widely developed due to the global prevalence of metabolic disorders like diabetes and obesity, which create a strong demand for personalized healthcare solutions. Like cardiac systems, these models have abundant data available, from biomarkers, lab tests, and wearable devices. These models hold significant potential for tailoring treatments by predicting individual responses to interventions such as medications, diet, and exercise.

The third most prevalent system modeled was the musculoskeletal system, for which most models were exclusively skeletal. This emphasis likely stems from the skeletal system’s foundational role in movement and load distribution, making it a logical starting point for biomechanical modeling. Moreover, bone structures are relatively straightforward to image and quantify using advanced modalities such as CT and MRI, facilitating accurate virtual reconstruction. Once imaged, bones can be modeled as mechanical structures with comparative ease, supporting the development of detailed and reliable simulations.

Interestingly, among the HDT models reviewed, no musculoskeletal models met the NASEM criteria for digital twins, and only a single cardiac model qualified. In contrast, eight of the 18 qualifying models (44.4%) were metabolic. Much of this can be attributed to the fact that only three unique metabolic models were identified across eight different studies. Metabolic models made up a disproportionately large share of NASEM-compliant digital twins—representing a threefold increase in relative representation compared to their ranking among all models included in the review. This overrepresentation of metabolic models among NASEM-compliant HDTs likely reflects both practical and conceptual factors. In many physiological systems, it is either difficult or unnecessary for the digital twin to directly influence its physical counterpart. The only fully autonomous HDT identified in this review was a cardiac model that modified the physical twin through automated defibrillator output. Outside of such specialized use cases, real-time updates to cardiac or musculoskeletal systems are more challenging to implement. For instance, while influencing heart rate may seem feasible in theory, implementing real-time adjustments to cardiovascular function typically requires indirect interventions and is less straightforward than metabolic modifications. A similar situation is realized when it comes to the musculoskeletal system, where an “update” to the physical twin conjures imagery of a robotic exoskeleton repositioning the patients’ limbs. On the other hand, updates to the metabolic system are easily realized in the form of recommendations regarding diet and exercise. Moreover, wearable devices such as continuous glucose monitors are comfortable, widely available, and capable of generating high-frequency data. By comparison, cardiac monitoring often relies on bulkier, less accessible tools (e.g. multi-lead ECGs or blood pressure cuffs), which may limit their integration into real-world, continuously updated HDT systems. Other organ systems, such as neurological or reproductive systems, are less represented likely due to lesser demand (relative to cardiac, metabolic, and musculoskeletal systems) and the reduced availability of high-quality data for model training and validation. Additionally, monitoring of these systems tends to be less straightforward than cardiac or metabolic systems. The technology required to perform such data collection, particularly in a continuous manner, may not be commonly available relative to devices such as wearable fitness trackers, portable blood pressure cuffs, or glucose monitors.

However, several emerging technologies may help address these limitations and expand the applicability of HDTs across a broader range of medical domains. In neurology, the growing adoption of wearable EEG headsets, brain-computer interface devices, and smart neurostimulation implants may soon enable continuous monitoring of brain activity in real-world environments. This could facilitate the development of digital twins for epilepsy management, neurodegenerative disease tracking, and cognitive function modeling. In reproductive health, advances in transdermal hormone sensors, smart fertility trackers, and implantable pelvic monitors could allow for dynamic modeling of ovulatory cycles, hormone fluctuations, and pelvic floor disorders. Beyond sensor hardware, advances in AI, particularly in multimodal data fusion and time-series forecasting, may allow researchers to synthesize heterogeneous data sources such as patient-reported outcomes, clinical notes, imaging studies, and wearable sensor data into cohesive, patient-specific digital models. This would decrease the amount of sensor data needed, placing more of an emphasis on dynamically updated patient history. Together, these innovations suggest that the delay in modeling of less common HDT systems may be temporary. As emerging technologies mature and become more widely accessible, it is likely that future HDT research will achieve broader system coverage, higher model fidelity, and greater clinical integration.

Despite the promise of HDTs to provide precision healthcare, the current study reveals several challenges to their development and classification. As per the inclusion criteria, all evaluated papers referred to the model at the center of their study as a digital twin. However, only a subset of these models met the definitional criteria outlined by the NASEM (Table 3). The factors necessary for compliance with the NASEM definition for digital twins are threefold. First, the system must be anchored by a mathematical model that represents the structure, behavior or function of a real-world object—in this context, the human body or one of its subsystems. Second, this mathematical model must be dynamically updated to reflect the evolving real-world circumstances of the physical object. In the present context, the model must be updated to reflect measured changes to the human body or one of its subsystems. Third, the model must possess predictive capabilities that inform the behavior or trajectory of the physical twin, ideally, to support goal-directed interventions or clinical decision-making. By our metrics, there was a single study^12^ that met the NASEM definition of a HDT in the traditional (i.e., engineering) sense, with the digital twin making automatic updates to the physical system to guide outcomes without external human input. Such systems are understandably rare, just as the conditions that warrant granting a device the unrestricted ability to effect physiological change to one’s body are rare. However, one such cardiac system was proposed by Lai et al.^12^. In their description, an implantable cardioverter defibrillator (ICD) would model a patient’s heart while monitoring for arrhythmias and restore normal function without human intervention. ICDs can provide timely therapies upon detection of irregular cardiac activity, but each individual patient requires personalized parameterization for these devices based on their personal cardiac history and device characteristics^12^. Furthermore, evolving conditions within singular patients necessitate that such parameters be adjusted regularly, though sometimes the need for such an adjustment can arise suddenly. The system proposed in Lai et al. would leverage ECG data to learn the patterns of a patient’s normal cardiac function and use this modeling as the basis upon which a digital twin of the patient’s heart was constructed. The digital twin monitored electrogram readings to detect arhythmic conditions. Upon discovery, it computes an appropriate electrical therapy to correct the cardiac dysfunction and then automatically administers the corrective action to restore the heart’s normal rhythm.

**Table 3:** Studies by model type

Model classification	Reference
Human digital twins	^12–29^
Traditional, automatic human digital twins	^12^
Human-in-the-loop human digital twins	^13–29^
Digital shadows	^30–43^
Personalized digital models	^6,7,44–128^
with one-time clinical decision support	^7,44–73^
without clinical decision support	^6,74–128^
General digital models	^129–143^
Virtual patient cohorts	^8,144–157^

The other category that would meet the NASEM standard of a true digital twin would be human-in-the-loop type twins, of which there were 17 studies^13–29^. Like the automatic twin systems, these systems generate recommendations to direct a patient’s health trajectory toward a particular goal. However, in these systems, a human—usually the patient or their physician—decides whether or not to follow these recommendations and may make modifications to the prescribed action as they see fit. An example of such a system was documented by Bahrami et al., whose reports described a digital twin that simulated oral and transdermal uptake of fentanyl based on a patient-specific parameterization of their computational model^14,22,24^. Real-world measurements reported by the physical twin included the patient’s respiration rate and self-reported pain score, which were used to update the digital counterpart. By targeting a fentanyl concentration range in the patient’s plasma, an acceptable breathing rate, and a minimal discomfort level, the digital twin could advise dosing schedules and delivery methods (e.g., when to change patches) for an optimized fentanyl administration strategy.

A second example of a human-in-the-loop style HDT was Twin Health’s twin precision nutrition/treatment program^18,26,27,29^. Traditional dietary guidelines for managing type 2 diabetes (T2D) are often based on population-level data and may not account for individual variation in metabolic responses. The digital model was constructed using a range of clinical parameters and continuously updated with data from multiple IoT-enabled devices. These included wearable fitness trackers (capturing steps, heart rate, sleep, and physical activity), a smart scale, an arm-based blood pressure monitor, a continuous glucose monitor, and a smartphone app used by patients to log their meals. The system then provided nutrition and lifestyle recommendations in tandem with registered dieticians working as health coaches to aid in the implementation of such recommendations.

While models like the Twin Precision system exemplify digital twins that offer real-time, personalized feedback to influence patient behavior, not all virtual representations of humans function in this way. Some instead are passive models, lacking interactivity or bidirectional influence—characteristics more aligned with digital shadows. The present study identified 14 such examples^30–43^. Like digital twins, digital shadows are parameterized models that receive input from the physical twin, which dictates the trajectory of the model—the main difference being that digital shadows do not make recommendations or updates to the physical system. Digital shadows comprise a virtual reflection of a system or object in the real world or, in the context of this report, a virtual reflection of a human body or subsystem. For example, a 2023 paper by Ou et al. described a system enabling the creation of a digital shadow of a patient which could be utilized in medical applications^37^. Their model used a photo to generate a model of the user’s face. Then, through the use of a camera and various tracking sensors, the system rendered a realistic, real-time representation of the user’s body in a virtual space. The rendering included simultaneous digital depictions of whole-body movements, facial expressions, and gaze direction. By faithfully conveying a patient’s posture and mental status, the team posited that the live avatar could constitute a virtual stand-in for telemedical applications or serve as a venue for life coaching systems for children with autism (potentially reducing social barriers in virtual peer interactions).

In contrast to digital shadows, which serve solely as passive reflections, personalized digital models are designed to simulate individualized physiology without requiring real-time updates. The present study identified 87 models fitting this category^6,7,44–128^. Personalized digital models share the individual customization of the digital shadows and the digital twins, but their parameters are not continuously updated by data from the physical twin. In some cases, these models are used to assess the current state of a patient and make a recommendation. In other cases, the model is created simply as a proof-of-concept or to gain an understanding of some aspect of the physical twin that is not easily observed using traditional instruments.

For example, a patient with a complex medical condition may face several possible treatment options. In such scenarios, a personalized computer model can be used to simulate each intervention and predict the likely outcome. Assuming the simulations are accurate, the physician and patient can select an appropriate therapy based on the predicted results. In these cases, once a successful intervention has been selected, further simulation is no longer required. The current study has identified 31 such models^7,44–73^. A study by O’Hara et al. exemplified personalized models offering one-time decision support^55^. In this system, a digital clone of a patient’s heart was generated from cardiac magnetic resonance data. The digital model was then used to predict abnormal electrical pathways in the patient’s heart that were causing irregular heartbeat rhythms (ventricular tachycardia circuits) for treatment via radio frequency ablation (RFA), a procedure that targets and destroys abnormal tissue to restore normal heart function. Once a successful ablation had been administered, there was no further need for the model and simulations were discontinued.

Beyond decision-support applications, many personalized digital models were developed for research, proof-of-concept, or physiological modeling purposes without directly informing clinical care. The present study identified 56 personalized models that did not inform treatment decisions^6,74–128^. An example of one such report is illustrated by Gillette et al.^76^. This study was a proof-of-concept demonstrating that a heart model suitable for use in a biophysically accurate HDT could be generated in a largely automatic fashion in a time frame deemed useable in a clinical setting. Using data from an MRI scan and a clinical, 12-lead ECG, their model captured the three-dimensional geometry of the heart, as well as a parameter vector specifying a comprehensive list of factors describing electrical processes which govern contraction of the lower heart chambers (ie ventricular activation and repolarization sequences). This model was not used to detect disease or inform a treatment strategy, though, in the future, it could be theoretically capable of synthesizing high fidelity ECG signals with near real time performance.

Cardiac models were represented in all types of models examined but were particularly prevalent as personalized digital models that were not used for decision support (*n* = 24). Oftentimes, these models aimed to mirror the cardiac physiology for a particular patient without providing output or recommendations. Either of the recently referenced examples of personalized digital models might constitute the foundation for a HDT if efforts were made to continually update each model with a parameter set reflective of the evolving conditions specific to the patient each model describes. Such is the case with most (if not all) of the models that have been presently categorized as personal models (cardiac or otherwise), though many of the situations reported upon did not require a full digital twin to achieve successful outcomes. Furthermore, many studies acknowledged that their model only constituted a digital twin insofar as their work represented a piece for inclusion within a larger digital twin project, whereby they contributed their piece to the digital twin puzzle.

While many personalized models are built around individual-level data, others lack this degree of specificity and are instead designed to represent average or theoretical human physiology. This study identified 15 examples of “digital twins” that are better described as general digital models^129–143^. Like the other systems detailed previously, general digital models are mathematical representations of—in our current context—humans or human subsystems. However, these model types are not specific to a particular individual. General digital models often predict outcomes that are statistically likely for a given population, but which will often diverge from the disease progression experienced by a particular individual. For example, the virtual liver model in Dichamp et al. contributed to the general understanding of liver processes, but did not aim to predict liver function in any specific individual^139^. In this paper, a mechanistic description of acetaminophen (APAP) hepatoxicity was presented which has allowed researchers to make reasonable extrapolations of APAP detoxification from laboratory measurements.

Some studies focused on creating simulated populations rather than representations of individual patients. These virtual patient cohorts serve as another distinct modeling approach commonly but inaccurately labeled as digital twins. The current study identified 15 examples of virtual patient cohorts^8,144–157^. A virtual patient cohort is a simulated group of patients generated via computational models and data to study disease patterns, treatment outcomes, or other medical scenarios without involving actual individuals. This approach allows researchers to explore and analyze various medical conditions and interventions in a controlled, virtual environment, often in-lieu-of or preliminary-to traditional clinical studies. For example, in a 2024 report Joslyn et al. described a 10-patient virtual cohort that was parameterized to reproduce experimental cellular kinetics observed in real patients^151^. The digital patients were then treated with different types of T cell therapies, and the analysis helped elucidate the biological processes governing such therapies in the body.

Beyond model classification, an important theme that permeates the NASEM report on digital twins is the assertion that the digital representations should be *fit for purpose*. The report stated that the technical details of the virtual representation—model types, fidelity, resolution, parameterization, and quantities of interest—must be selected to align with the specific decision task and computational constraints. While early conceptualizations of digital twins often emphasized high-fidelity, physics-based simulations, current thinking is more pragmatic: what matters most is that the model fulfills its intended purpose, regardless of its underlying structure. The models included in the present study featured a wide range of complexity, from rules-based systems to mechanistic models simulating the underlying physics. The distribution of model types showed a slight preference for empirical approaches (42.95%), followed by mechanistic (31.55%) and hybrid (25.50%) models. While empirical models were somewhat overrepresented, the overall balance among the three categories remained relatively even. Among the models meeting the NASEM criteria for digital twins, the distribution shifted further in favor of empirical approaches. These comprised the majority (61.11%), followed by mechanistic (22.22%) and hybrid models (16.67%). In this category, a disproportionately large number of the studies consisted of multiple reports on what is probably the same model. When considering a count of likely unique models, empirical models comprised 66.66%, with hybrid and mechanistic models splitting the remainder (16.67% each).

In addition to the complexity and fidelity of the models, the sensors used to inform their digital counterparts similarly varied. Across the full set of included studies, 23.5% of studies used consumer grade sensors. However, when the focus was narrowed to only the digital twins meeting the NASEM criteria, the portion that used consumer grade sensors increased to 55.6%. Conversely, in the total population of included studies, the portion that employed clinical grade sensors was 61.07%, while the portion in the NASEM digital twin group decreased to 27.8%. This trend remained consistent even after adjusting for likely unique models. In that context, the proportion of consumer-grade sensors rose from 21.9% to 50.0% when shifting from the broader population to NASEM-compliant models, while clinical-grade sensor use decreased from 62.8% to 41.7%. This may reflect the need for portable sensors for the regular updates required by HDTs, whereas clinical sensors may be too large, expensive, cumbersome, and/or impractical for subjects to carry and maintain. This could be indicative of an outsized role that consumer grade devices may play in the evolving HDT research space.

In addition to model design and data input considerations, the rigor with which digital twin models are evaluated is critically important. In their report on digital twins, NASEM stressed the importance of verification, validation, and uncertainty quantification (VVUQ) for all aspects of digital twin research. In summary, verification involves checking that a computer program correctly implements and solves the equations of a mathematical model, including code verification and solution verification. That is, verification exists to ensure the computer programming correctly performs intended algorithms while assessing the accuracy of its solutions. Validation is about evaluating how accurately a model represents the real world with respect to its intended usage. Uncertainty quantification focuses on measuring incertitude in model calculations, with an aim to account for all sources of uncertainty and quantify the impact of each on the overall results. The nature of HDTs does not render researchers exempt from VVUQ considerations: the NASEM report specifically lists a cancer patient twin as an example scenario for which VVUQ protocols should be applied. In the present work, only two papers of all included studies contained the term VVUQ (in the body of the text). While both of these works conducted their studies in the spirit of VVUQ, they both pointed to reasons why a rigorous treatment of VVUQ standards cannot be satisfactorily applied at present. One^64^ highlighted the absence of a clear pathway for VVUQ in the context of obtaining regulatory approval for in silico clinical trials and digital patients. Roughly two thirds of included studies had some treatment of uncertainty and/or model validation, but a small subset of these addressed many of the VVUQ concerns without explicitly mentioning the term (e.g., refs. ^57,88,94,126,128^).

The importance of VVUQ in digital twin development cannot be overstated, particularly in healthcare, where model accuracy directly impacts clinical decision-making. For researchers wishing to employ a rigorous treatment of VVUQ, the American Society of Mechanical Engineers (ASME) Validation and Verification (V&V) Standards Committee in Computational Modeling and Simulation has released a suite of standards that can be adopted or serve as examples for HDT endeavors^159–161^. Additionally, the 2012 NRC report on VVUQ provides comprehensive guidance for those wishing to incorporate such practices into their work^11^.

Teams working on patient specific models for medical applications are already leveraging VVUQ methodology, and they can provide valuable insights into how VVUQ can be systematically assimilated into HDT research. For instance, Galappaththige et al. outline a comprehensive roadmap for incorporating VVUQ practices into the development of personalized digital models^162^. This team instructively demonstrates adherence to the ASME V&V 40 (2018) standards, which provide a robust framework for assessing model credibility in medical device applications. Similarly, the work of Santiago et al.^163^ and Nagaraja et al.^164^ showcase detailed strategies toward the design and execution of robust VVUQ plans, exemplifying how standardized methodologies can enhance model reliability and stakeholder confidence. Building on these examples, HDT researchers can develop these practices by leveraging established standards to define specialized VVUQ tailored to HDT applications.

The implementation of VVUQ in HDT studies will necessarily vary based on the model’s purpose, design, and clinical context. Mechanistic HDTs, which rely on physics- and math-based representations of human systems, require rigorous verification and validation to ensure that their theoretical frameworks are both accurate and adaptable to individual patients. These models must be customized to capture patient-specific parameters and reliably predict how different interventions will influence physiological outcomes. In contrast, empirically trained HDTs, often powered by machine learning, must focus on the adequacy and representativeness of their training data. These models require robust validation on external datasets and careful uncertainty quantification to ensure that their predictions generalize accurately to the human systems they aim to model. The degree of VVUQ stringency required also depends on whether the HDT outputs are interpreted by a human or implemented automatically. Human-in-the-loop systems can tolerate a higher degree of uncertainty because human judgment provides an additional safety layer. Conversely, HDTs that autonomously update the physical twin, such as implantable devices that adjust physiological parameters in real time, must meet far more stringent VVUQ standards to ensure patient safety. In these settings, model outputs are acted upon without human oversight, necessitating high confidence in model reliability and robustness. For instance, metabolic HDTs that provide lifestyle recommendations may tolerate some level of predictive uncertainty, as their outputs pose relatively low immediate risk. In contrast, cardiac HDTs that influence real-time hemodynamic parameters demand significantly lower uncertainty thresholds to prevent harm. Thus, the level of trust required, and the methods used to establish that trust must be tailored to the clinical application and technical design of the HDT.

Despite offering a comprehensive overview of the HDT landscape, this review is subject to several limitations that warrant consideration. While the categories discussed in this review are well-defined, the boundaries between categories can be unclear. This imprecision leads to a degree of subjectivity in categorizing and classifying the models and concepts reviewed. The subjective nature of these categorizations may influence the consistency and comparability of the results. Additionally, the categorization of models was retrospective and determined by the discretion of the authors to best summarize the body of publications regarding HDTs. To the authors’ knowledge, there are no well-established categorization systems for HDTs. As such, some studies may have fit into multiple categories with regard to model type but were placed into the one single category which was considered to be the best fit.

Additionally, this review is limited by the quality of the included studies. Concerns about study quality often center around methodological rigor and the validity of treatment outcomes. Since the present review is focused on the usage and interpretation of specific terminology, as opposed to the evaluation of medical interventions, the impact of study quality is somewhat diminished. Although variations in reporting standards and analytical approaches exist, these issues are less likely to skew the findings in the context of our analysis. Our primary concern is an assessment of how the term “digital twin” is defined and used across different studies, which, while important, does not carry the same weight as methodological issues would in a clinical review. Therefore, while the quality of studies remains a consideration, its relative impact on our conclusions is minimal compared to that in traditional systematic reviews. Furthermore, this team is unequipped to evaluate the general language competency of each group in a way that would be relevant to the present research, and any assessment we could perform would be highly subjective and of questionable value. Indeed, many of the typical measures of quality (e.g., bias, validity, sample size, study design, data collection methods) are all irrelevant to the context surrounding how each group used the term “digital twin”, so the measures of quality that should be employed in the present case are uncertain. In light of these considerations, the choice was made to not include a formal quality assessment.

Despite a lack of formal quality assessment, we observed that most studies successfully achieved their stated objectives—often focusing on evaluating model performance, even if the models themselves did not qualify as HDTs. However, methodological rigor varied considerably across the literature. Some studies demonstrated strong practices, such as incorporating real-world patient data, integrating multimodal sources, and clearly articulating their modeling rationale. Mechanistic studies often excelled at generating generalized models grounded in first principles. However, these models were typically the farthest from meeting the criteria for digital twins, as they lacked personalization or dynamic interaction with a physical counterpart. Empirical studies, on the other hand, were often methodologically sound in their approach to model development and evaluation—frequently dividing their data into training and testing sets and, in some cases, providing access to source code or datasets to support reproducibility. Despite these strengths, many studies lacked external validation, detailed reporting on reproducibility measures, or comprehensive approaches to uncertainty analysis. Empirical models were typically validated using held-out portions of the training data, while mechanistic studies were often limited to proof-of-concept demonstrations. More robust mechanistic models tested their predictions against real-world data, though such efforts were less common. This variation highlights the evolving nature of HDT research and reinforces the need for more standardized reporting, validation frameworks, and methodological transparency.

While variability in methodological rigor was evident, the findings of this review offer important insight into current usage patterns and definitional inconsistencies in the HDT literature. In our scoping review of publications including the term “digital twin” since 2017, we found that 18 out of 149 studies (12.1%) appropriately used the term in describing their models, while the remainder (87.9%) applied the term to models that did not align with the NASEM definition of a digital twin. Pervasive misuse of the term “human digital twin” confuses and complicates future efforts to advance healthcare.

Establishing a solid foundation for the term “human digital twin” requires setting clear standards for its usage. In order to effectively implement this concept, the health research community must embrace the framework set forth by the NASEM digital twin report. Going forward, the computational models appearing in HDT studies should incorporate three essential elements:

(1) a digital model parameterized to be representative of a specific individual.

(2) dynamic, ongoing updates to the digital twin model with data collected by sensors (etc.) reporting measurements quantifying the state of the specific individual (or part thereof) that the digital twin model represents.

(3) ongoing modification to the state or behavior of said specific individual, based on insights and predictions generated by the digital twin model.

**Fig. 2:**
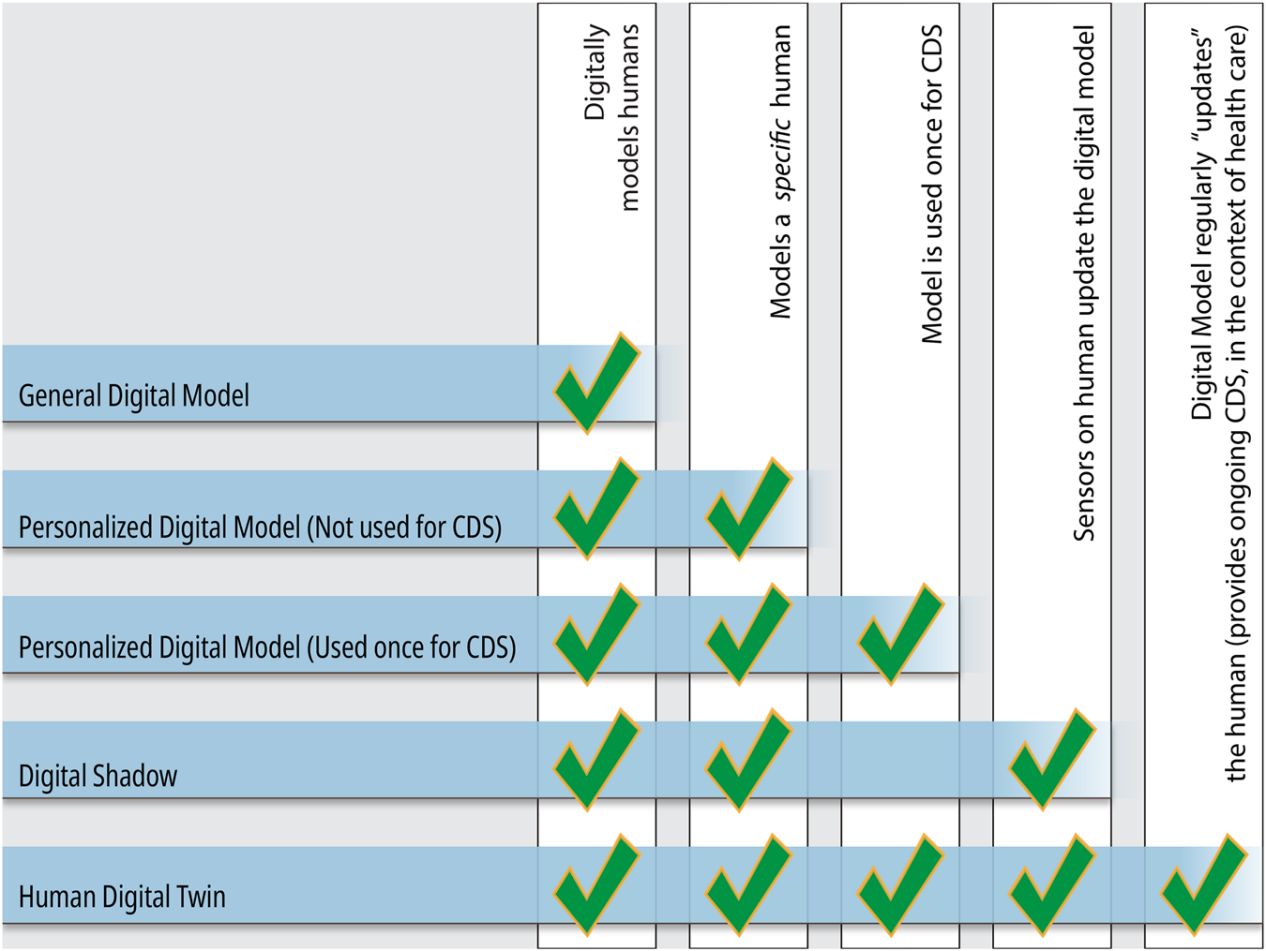
Properties of the various model types. Digital model is an all-encompassing term under which most, if not all, computerized models can be categorized. When a digital model is tailored to reflect the unique circumstances pertaining to a specific individual, they become *personalized* digital models. If a personalized digital model continually accepts input from the individual and updates itself accordingly, then the model can be considered a digital shadow. Digital shadows, however, are passive and do not alter the state or trajectory of the individual being modeled, merely providing a digital reflection of the individual. On the other hand, if a model that would otherwise be categorized as a digital shadow provides some form of predictive output that automatically makes decisions that affect the individual, or if it provides information that said individual (or their care team) uses to inform decisions affecting the individual (e.g. which treatment option to take, or how vigorously to exercise), then the model can be considered a human digital twin. Many of the personalized digital models examined herein were used to inform a single decision or treatment option for the individual, so the present study makes the distinction between personalized models that were used once for clinical decision support (CDS) and those that were never used to alter the individual’s trajectory. For a “used-once” personalized model to be considered a human digital twin, it would require that the model be updated after each decision was executed in order to inform additional decisions, in a continual, ongoing process. The personalized digital models that were not used for CDS are like digital shadows, except they only capture one moment in time, whereas the digital shadow will dynamically reflect the individual’s state in an ongoing process.

**Fig. 3:**
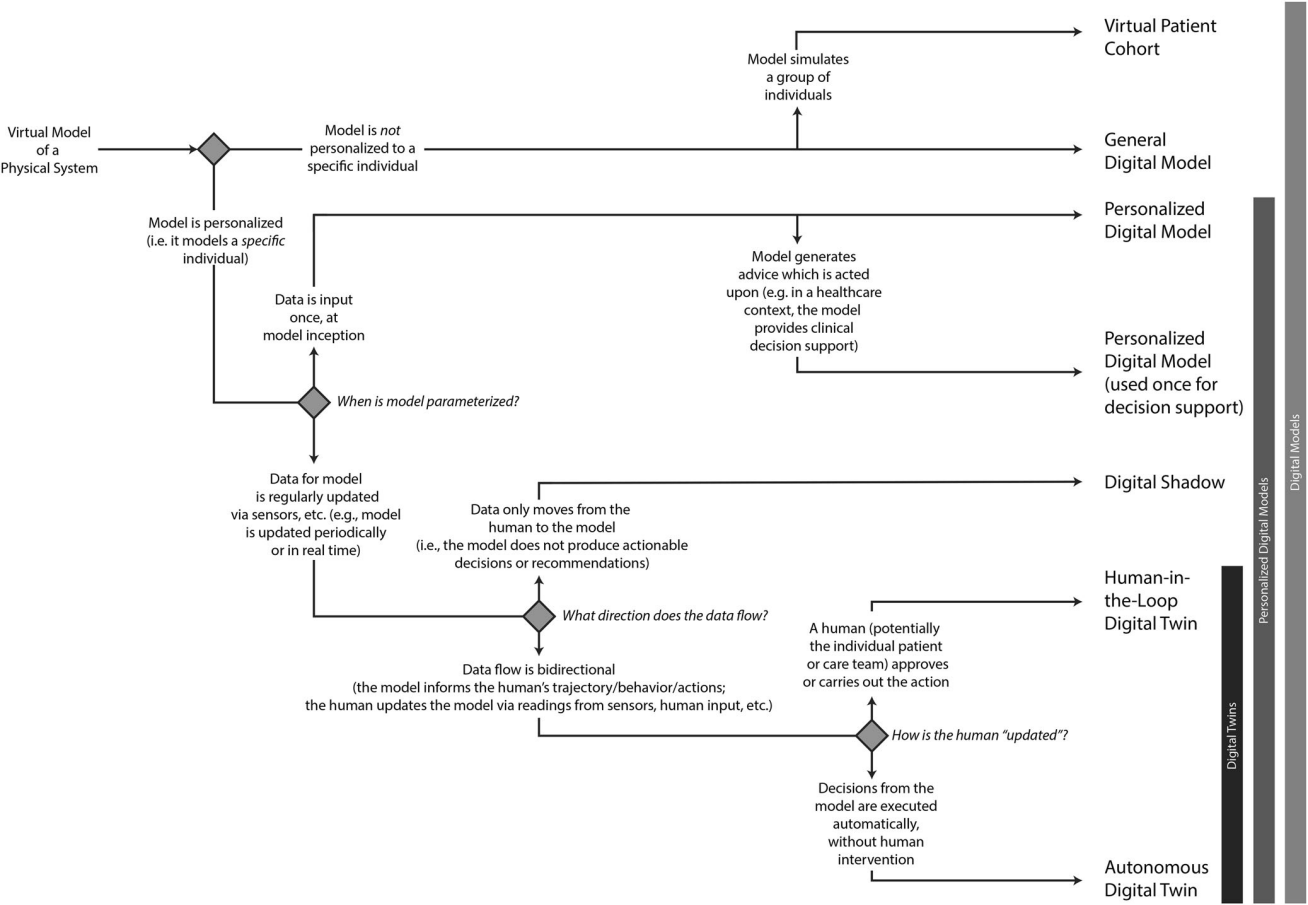
Taxonomic flow chart for digital models. This flow chart can be used to determine the categorization of digital models, differentiating between virtual patient cohorts, general digital models, personalized digital models, digital shadows and human digital twins.

Models which do not meet the definition of a HDT, such as digital shadows, general digital models, and virtual patient cohorts, should be categorized correctly and consistently in literature as well (Figs. 2 and 3).

**Fig. 4:**
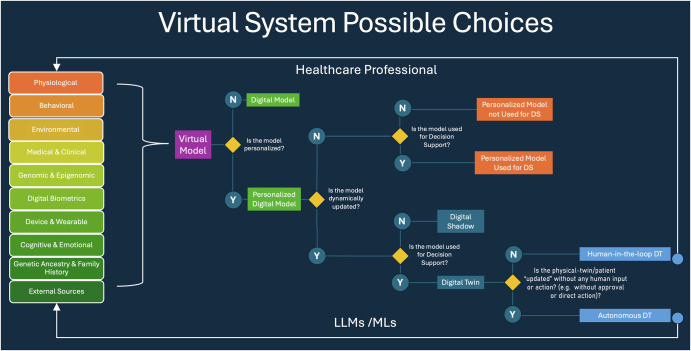
Model classification and data flow for digital models—differentiating digital models from digital shadows and human digital twins, in healthcare. Data flows into a virtual model from a variety of sources, stemming from human subjects or their environment. Some models are parameterized exactly once, while others are dynamically updated in a recurring fashion. In digital twin models, the data flows from the model back into the data-sources (i.e. the subject or physical system is somehow modified based on predictions made by the digital twin) such that the ongoing model parameterization reflects decisions and advice generated by the model.

This comprehensive approach will ensure that usage of the term “human digital twin” is both consistent and accurate, setting a precedent for its future development and application in healthcare. This work provides a framework to systematically and rigorously categorize studies involving digital models and HDTs in healthcare, ensuring a more precise alignment with their conceptual definitions and promoting consistency in their scholarly classification (Figs. 3 and 4).

To address the need for standardization and clearer definitions in the HDT field, we propose a forward-looking roadmap organized into short- and long-term goals. In the short term, researchers should focus on adopting standardized terminology by promoting consistent use of the terms “digital twin” and “human digital twin” in alignment with NASEM criteria, and by clearly distinguishing related concepts such as general digital models, personalized digital models, digital shadows, and virtual patient cohorts. While comprehensive VVUQ may not be feasible in early-stage studies, researchers should aim to transparently report validation steps, modeling assumptions, and sources of uncertainty. Early interdisciplinary collaboration among academic, clinical, and industry stakeholders will also be critical to ensure that models are clinically relevant and grounded in real-world utility throughout their development. Finally, to improve reporting standards and methodological rigor, journals and funding bodies should encourage authors to provide clear descriptions of model architecture, data inputs, training and testing protocols, and performance evaluation metrics. Encouraging transparency in training data provenance and parameterization practices will further support reproducibility and facilitate meaningful comparison between models.

Over the longer term, more rigorous infrastructure and guidance will be needed to support scalable, trustworthy HDT deployment. This includes the development and adoption of HDT-specific VVUQ frameworks, potentially by adapting existing standards such as ASME V&V 40 to account for patient variability, real-time model updates, and clinical risk. Regulatory bodies such as the Food and Drug Administration and European Medicines Agency should be engaged to define approval pathways and evaluation criteria specific to HDTs. To support reproducibility and collaboration, shared infrastructure is also needed, including open-access data repositories, benchmark modeling tasks, and shared validation datasets. Finally, clinical implementation studies should be prioritized to evaluate how HDTs integrate into real-world workflows and influence decision-making, safety, and patient outcomes. By gradually integrating these actionable steps, a solid foundation is laid for the widespread implementation and efficacy of HDTs in healthcare.

## Methods

A review protocol for this study was not registered.

### Search strategy and selection criteria

We devised a systematic search to identify research regarding HDTs in healthcare, conducted on the PubMed, Embase, and IEEE Xplore databases. These databases were selected to obtain a comprehensive search of digital twins in both medical and biomedical engineering applications and the following queries were executed on July 3rd, 2024:

Pubmed: <2017 – 2024 week 27>

("digital twin*"[Title/Abstract] AND ("medic*"[Title/Abstract] OR "medicine"[MeSH Terms] OR medicine OR "health*"[Title/Abstract] OR "health"[MeSH Terms] OR health OR "patient*"[Title/Abstract] OR "patients"[MeSH Terms] OR patient OR "hospital*"[Title/Abstract] OR "hospitals"[MeSH Terms] OR "clinic*"[Title/Abstract]) AND (2017:2024[pdat]))

Embase: <2017 – 2024 week 27>

('digital twin':ti,ab,kw OR 'digital twin':de) AND ('medic*':ti,ab,kw OR 'medicine'/exp OR medicine OR 'health*':ti,ab,kw OR 'health'/exp OR health OR 'patient*':ti,ab,kw OR 'patient'/exp OR patient OR 'hospital*':ti,ab,kw OR 'hospital'/exp OR hospital OR 'clinic*':ti,ab,kw OR 'clinic'/exp OR clinic) AND [2017-2024]/py

IEEE Explore: <2017 – 2024 week 27>

("Document Title":"digital twin*" OR "Document Abstract":"digital twin*" OR "Author Keywords":"digital twin") AND (("Document Title":"medic*" OR "Document Abstract":"medic*" OR "Author Keywords":"medic*" OR "Mesh_Terms":"Medicine") OR ("Document Title":"health*" OR "Document Abstract":"health*" OR "Author Keywords":"health*" OR "Mesh_Terms":"Health") OR ("Document Title":"digital twin*" OR "Document Abstract":"digital twin*" OR "Author Keywords":"digital twin") AND ("Document Title":"patient*" OR "Document Abstract":"patient*" OR "Author Keywords":"patient*" OR "Mesh_Terms":"patient") OR ("Document Title":"hospital*" OR "Document Abstract":"hospital*" OR "Author Keywords":"hospital*" OR "Mesh_Terms":"hospitals") OR ("Document Title":"clinic*" OR "Document Abstract":"clinic*" OR "Author Keywords":"clinic*"))

We limited searches to peer-reviewed studies published after January 1st, 2017, following a review by Katsoulakis et al. which showed the publication of articles referring to digital twins in healthcare has become more prevalent since 2017^3^. Search terms included “Digital Twin*” and any of the following: “medic*”, “health*”, “patient*”, “hospital*”, or “clinic*”. We further enhanced the results by manually examining citations and reference lists and tracking citations of relevant publications throughout the review process. We limited the search to English-language articles. We reviewed all studies reporting on HDTs for inclusion. However, the final selection focused on studies reporting to have either created or evaluated HDTs, including those that created or evaluated “digital twins” of a human system (e.g., organ systems, cellular structures, or body parts). We included studies that claimed to create HDTs, even if the digital twin was not the focus of the article. We excluded studies that presented frameworks for the technical creation of digital twins but did not demonstrate use cases for their implementation. We also excluded studies that claimed to create digital twins but did not create *human* digital twins (i.e., studies that claimed to create or evaluate digital twins of non-human objects or systems). We excluded letters to the editor, abstracts without full texts, commentaries, editorials, and review articles.

Exclusion of review articles was rooted in our desire to focus on primary data and applications. Additionally, we aimed to avoid redundancy that could arise if review authors adopted the terminology used by their reviewees. For example, a reviewer might refer to a NASEM HDT as a ‘digital twin’ in one project and to a digital shadow as a ‘digital twin’ in another project, simply because those were the terms used by the respective reviewees. To avoid the potential ambiguities and redundant classifications, the decision was made to exclude review articles.

Similarly, framework papers posited about how certain digital twins might be achieved, but without a tangible model for evaluation our ability to reliably judge and categorize a team’s specific implementation falters. Frameworks frequently focused on a few key, novel aspects of their proposed models, providing detailed descriptions of these components. However, they often neglected to address the practical implementation or use of their ‘digital twins,’ which is essential for evaluating compliance with the NASEM definition. In an effort to avoid ambiguity with frameworks whose categorization was contingent upon details that were not precisely articulated, we opted to exclude frameworks altogether for the sake of consistency.

### Study selection and data extraction

We extracted and deduplicated database search results. Two independent reviewers (RS and BT) performed title and abstract screening on Covidence^165^, with conflicts resolved by consensus. Articles were then sought for full-text access and review. The independent reviewers (RS and BT) performed a full text review, with conflicts for inclusion resolved by consensus. We utilized Microsoft Excel to extract study characteristics (author affiliation, classification of the model, design of model, and type of digital twin). An independent reviewer (RS) extracted data, and another reviewer (BT) assessed data for accuracy and rectifying inconsistencies.

### Author affiliation

We defined author affiliation as the institution associated with the corresponding author and was grouped as academic/medical, industry, or government.

### Model design

We categorized the design of the model as empirical, mechanistic, or hybrid. Empirical models of digital twins included models that utilized data to train machine learning (ML) models for predictive purposes and models that otherwise identified trends in measured data through techniques such as regression or traditional statistical analyses. Mechanistic models included those that relied on first principles for their development—models computing their predictions based upon the underlying chemistry or physics, as opposed to statistics or curve fitting—allowing patient-specific personalization through the adjustment of pre-determined parameters to customize the model. Hybrid models utilized elements of both model types to customize the “twin” to the specific patient.

### Model classification

We determined the classification of the model based on the NASEM definition of digital twins and the use of patient-specific personalization, dynamic updating of input and output parameters, and whether the model was intended to be utilized for decision support. We made a further distinction between a model’s production of human-in-the-loop recommendations versus automatic updates to the human system. Thus, after conducting a preliminary review of literature, we stratified the models into the following categories: virtual patient cohort, digital model, personalized digital model, personalized digital model used once for decision support, digital shadow, human-in-the-loop digital twin, and traditional digital twin. Although distinct entities, human-in-the-loop and traditional HDTs are both considered to satisfy the NASEM criteria for digital twins. A detailed description of different model types can be found in Figs. 2–4.

A general digital model involves the digital representation of a class of real-world objects, without automatic data updates from physical to virtual and without being specific to a particular object—or in the present case, without being specific to a particular person^4^. For example, a general digital model of a liver may predict probable metabolism of a specific drug. A personalized digital model may expand on this to predict liver drug metabolism for a particular patient utilizing their specific liver enzyme activity. A personalized digital model utilized once for decision support may go further to predict which medication a patient in liver failure should take in a one-time use case. Please note that the term “patient specific model (PSM)” is often used in this sense, but in the present study we choose to use the more general term “personalized digital model” in order to be inclusive of applications where the subject is not, strictly speaking, a patient (e.g., fitness and lifestyle models), though it is understood that many of the personalized digital models described herein would technically be better categorized as PSMs. A digital shadow involves a dynamic one-way data flow between the state of an existing physical object and a digital object^4^. In the example of a liver, the patient may obtain labs every week to personalize their liver model over time; such dynamic updates reflect their current changes in liver enzymes and metabolism. A digital shadow simply seeks to model a changing system and does not inform decisions. A dynamically updated personalized liver model that provides updated medication recommendations with each information update would meet the NASEM definition of a digital twin. Such recommendations would involve a human-in-the-loop to dispense the medication. If, hypothetically, the patient had a device to provide medication to their blood directly, this would be considered a digital twin with automatic updates and would be closest to the definition of a traditional digital twin. Additionally, if the term digital twin was referring to the creation of several distinct iterations of the patient’s liver with different parameters to test possible responses to a medication, this would more so refer to a virtual patient cohort, which has been previously described in literature^166^.

### Categorization of digital models

We retrospectively identified 13 categories of digital models depending on the system they aimed to describe. These included cardiac, musculoskeletal, respiratory, neurological, hepatic, metabolic, epidermal, reproductive, immune, cancer, whole body, surgical site, and other twins. Metabolic twins were further broken down into diabetic twins if they attempted to model the metabolism in patients with diabetes. Musculoskeletal twins were further classified as skeletal twins if they focused solely on skeletal structures.

### Sensor classification

We recorded a tabulation regarding clinical grade versus consumer grade measurements for model creation and updates. Data taken from devices that are typically only available in a clinical setting were categorized as clinical grade. Examples include measurements from 12-lead ECG data, MRI data, or CT scans. Measurement data from devices that are available from retail outlets or are publicly available were classified as consumer grade. In this category, examples include smart phones, wearable fitness trackers (e.g., Fitbit Sense 2), consumer video signals (e.g., Microsoft Kinect, web cams), scales (smart or otherwise), and subjective, self-reported scores (e.g., pain scores). Additionally, we recorded and categorized imaging data collected by clinical and consumer grade devices by modality—including video data, still photographs, MRI data, CT data, radiographic imaging, and other imaging systems.

### Verification, validation, and uncertainty quantification (VVUQ)

We screened studies for mention of the VVUQ process. We further analyzed studies that included information regarding VVUQ for adherence to VVUQ standards via self-reporting.

### Data analysis

We narratively synthesized and presented descriptively data as frequencies and percentages. We conducted cross-tabulations to further stratify and describe the model classification.

## Supplementary information

Supplementary Information

## Data Availability

The datasets generated and analyzed during the current study are available upon reasonable request from the corresponding author.

## References

[1] Grieves, M. & Vickers, J. Digital twin: mitigating unpredictable, undesirable emergent behavior in complex systems. In *Transdisciplinary Perspectives on Complex Systems: New Findings and Approaches.* (eds Kahlen, F.-J., Flumerfelt, S. & Alves, A.) 85–113 (Springer International Publishing, 2017).

[2] Glaessgen, E. & Stargel, D. The digital twin paradigm for future NASA and U.S. Air Force Vehicles. In *53rd AIAA/ASME/ASCE/AHS/ASC Structures, Structural Dynamics, and Materials Conference.* Reston, VA (AIAA, 2012). 10.2514/6.2012-1818.

[3] Katsoulakis, E. et al. Digital twins for health: a scoping review. *NPJ Digital Med.* **7**, 77 (2024). 10.1038/s41746-024-01073-0 PMC10960047 38519626

[4] Kritzinger, W., Karner, M., Traar, G., Henjes, J. & Sihn, W. Digital twin in manufacturing: a categorical literature review and classification. *IFAC-PapersOnLine* **51**, 1016–1022 (2018).

[5] Shengli, W. Is human digital twin possible? *Comput. Methods Prog. Biomed. Update* **1**, 100014 (2021).

[6] Jin, Z. et al. Anti- and pro-fibrillatory effects of pulmonary vein isolation gaps in human atrial fibrillation digital twins. *NPJ Digital Med.* **7**, 81 (2024). 10.1038/s41746-024-01075-y PMC10966060 38532181

[7] Mösch, A., Grazioli, F., Machart, P. & Malone, B. NeoAgDT: optimization of personal neoantigen vaccine composition by digital twin simulation of a cancer cell population. *Bioinformatics* **40**, 1–9 (2024). 10.1093/bioinformatics/btae205 PMC11076149 38614133

[8] Chasseloup, E., Hooker, A. C. & Karlsson, M. O. Generation and application of avatars in pharmacometric modelling. *J. Pharmacokinet. Pharmacodyn.* **50**, 411–423 (2023). 37488327 10.1007/s10928-023-09873-9 PMC10460751

[9] Knab, T. D., Clermont, G. & Parker, R. S. A “virtual patient” cohort and mathematical model of glucose dynamics in critical care. *IFAC-PapersOnLine.* **49**, 1–7 (2016).

[10] National Academies of Sciences, Engineering, and Medicine. *Foundational Research Gaps and Future Directions for Digital Twins. Washington, DC: The National Academies Press*. 10.17226/26894 (2024). 39088664

[11] National Research Council. *Assessing the Reliability of Complex Models: Mathematical and Statistical Foundations of Verification, Validation, and Uncertainty Quantification.* (The National Academies Press, 2012).

[12] Lai, M., Yang, H., Gu, J., Chen, X. & Jiang, Z. Digital-twin-based online parameter personalization for implantable cardiac defibrillators. *Annu. Int. Conf. IEEE Eng. Med. Biol. Soc.* **2022***,* 3007–3010 (2022). 10.1109/EMBC48229.2022.9871142 36086607

[13] Chakshu, N. K. & Nithiarasu, P. An AI based digital-twin for prioritising pneumonia patient treatment. *Proc. Inst. Mech. Eng. H.* **236**, 1662–1674 (2022). 36121054 10.1177/09544119221123431 PMC9647318

[14] Bahrami, F., Rossi, R. M., De Nys, K. & Defraeye, T. An individualized digital twin of a patient for transdermal fentanyl therapy for chronic pain management. *Drug Deliv. Transl. Res.* **13**, 2272–2285 (2023). 36897525 10.1007/s13346-023-01305-y PMC10382374

[15] Laamarti, F. et al. An ISO/IEEE 11073 standardized digital twin framework for health and well-being in smart cities. *IEEE Access* **8**, 105950–105961 (2020).

[16] Lal, A. et al. Development and verification of a digital twin patient model to predict specific treatment response during the first 24 h of sepsis. *Crit. Care Explor.* **2**, e0249 (2020). 33225302 10.1097/CCE.0000000000000249 PMC7671877

[17] Maruyama, T. et al. Digital twin-driven human robot collaboration using a digital human. *Sensors* **21**, 8266 (2021). 34960355 10.3390/s21248266 PMC8709080

[18] Joshi, S. et al. Digital twin-enabled personalized nutrition improves metabolic dysfunction-associated fatty liver disease in type 2 diabetes: results of a 1-year randomized controlled study. *Endocr. Pract.* **29**, 960–970 (2023). 37778441 10.1016/j.eprac.2023.08.016

[19] Pellizzari, E., Prendin, F., Cappon, G., Sparacino, G. & Facchinetti, A. drCORRECT: an algorithm for the preventive administration of postprandial corrective insulin boluses in type 1 diabetes management. J. Diabetes Sci. Technol. 19322968231221768 10.1177/19322968231221768 (2023). 10.1177/19322968231221768 PMC12035290 38158565

[20] Béthencourt, L. et al. Guiding measurement protocols of connected medical devices using digital twins: a statistical methodology applied to detecting and monitoring lymphedema. *IEEE Access* **9**, 39444–39465 (2021).

[21] Thamotharan, P. et al. Human digital twin for personalized elderly type 2 diabetes management. *J. Clin. Med.* **12**, 2094 (2023). 36983097 10.3390/jcm12062094 PMC10056736

[22] Bahrami, F. et al. Implementing physics-based digital patient twins to tailor the switch of oral morphine to transdermal fentanyl patches based on patient physiology. *Eur. J. Pharm. Sci.* **195**, 106727 (2024). 38360153 10.1016/j.ejps.2024.106727

[23] Tardini, E. et al. Optimal treatment selection in sequential systemic and locoregional therapy of oropharyngeal squamous carcinomas: deep Q-learning with a patient-physician digital twin dyad. *J. Med. Internet Res.* **24**, e29455 (2022). 35442211 10.2196/29455 PMC9069283

[24] Bahrami, F., Rossi, R. M. & Defraeye, T. Predicting transdermal fentanyl delivery using physics-based simulations for tailored therapy based on the age. *Drug Deliv.* **29**, 950–969 (2022). 35319323 10.1080/10717544.2022.2050846 PMC8956318

[25] Abeltino, A. et al. Putting the personalized metabolic avatar into production: a comparison between deep-learning and statistical models for weight prediction. *Nutrients.* **15**, 1199 (2023). 36904199 10.3390/nu15051199 PMC10004838

[26] Shamanna, P. et al. Reducing HbA1c in type 2 diabetes using digital twin technology-enabled precision nutrition: a retrospective analysis. *Diab. Ther.* **11**, 2703–2714 (2020). 10.1007/s13300-020-00931-w PMC7547935 32975712

[27] Shamanna, P. et al. Retrospective study of glycemic variability, BMI, and blood pressure in diabetes patients in the Digital Twin Precision Treatment Program. *Sci. Rep.* **11**, 14892 (2021). 34290310 10.1038/s41598-021-94339-6 PMC8295289

[28] Cappon, G. et al. System architecture of TWIN: a new digital twin-based clinical decision support system for type 1 diabetes management in children. In *Proc. 2023 IEEE 19th International Conference on Body Sensor Networks (BSN).* 1–4 (IEEE, 2023). 10.1109/BSN58485.2023.10331272.

[29] Shamanna, P. et al. Type 2 diabetes reversal with digital twin technology-enabled precision nutrition and staging of reversal: a retrospective cohort study. *Clin. Diab. Endocrinol.* **7**, 21 (2021). 10.1186/s40842-021-00134-7 PMC8591797 34776010

[30] Gorelova, A., Meliá, S. & Gadzhimusieva, D. A discrete event simulation of patient flow in an assisted reproduction clinic with the integration of a smart health monitoring system. *IEEE Access* **12**, 46304–46318 (2024).

[31] Taneja, K. et al. A feature-encoded physics-informed parameter identification neural network for musculoskeletal systems. *J. Biomech. Eng.* **144**, 121006 (2022). 35972808 10.1115/1.4055238 PMC9632475

[32] Khan, S., Alzaabi, A., Iqbal, Z., Ratnarajah, T. & Arslan, T. A novel digital twin (DT) model based on WiFi CSI, signal processing and machine learning for patient respiration monitoring and decision-support. *IEEE Access* **11**, 103554–103568 (2023).

[33] Tanade, C., Rakestraw, E., Ladd, W., Draeger, E. & Randles, A. Cloud computing to enable wearable-driven longitudinal hemodynamic maps. In *Proc. International Conference for High Performance Computing, Networking, Storage and Analysis (SC23).* (ACM, 2023). 10.1145/3581784.3607101. 10.1145/3581784.3607101 PMC11210499 38939612

[34] Uhlenberg, L., Derungs, A. & Amft, O. Co-simulation of human digital twins and wearable inertial sensors to analyse gait event estimation. *Front. Bioeng. Biotechnol.* **11**, 1104000 (2023). 37122859 10.3389/fbioe.2023.1104000 PMC10132030

[35] Riedel, P., Riesner, M., Wendt, K. & Aßmann, U. Data-driven digital twins in surgery utilizing augmented reality and machine learning. In *Proc. 2022 IEEE International Conference on Communications Workshops (ICC Workshops).* 580–585 (IEEE, 2022). 10.1109/ICCWorkshops53468.2022.9814537.

[36] Batch, K. E. et al. Developing a cancer digital twin: supervised metastases detection from consecutive structured radiology reports. *Front. Artif. Intell.* **5**, 826402 (2022). 35310959 10.3389/frai.2022.826402 PMC8924403

[37] Ou, H. et al. Development of a low-cost and user-friendly system to create personalized human digital twin. In *Proc. 45th Annual International Conference of the IEEE Engineering in Medicine and Biology Society (EMBC).* 1–4 (IEEE, Sydney, Australia, 2023). 10.1109/EMBC40787.2023.10340461. 10.1109/EMBC40787.2023.10340461 38082694

[38] Guo, Y. et al. Digital twin-driven dynamic monitoring system of the upper limb force. *Comput. Methods Biomech. Biomed. Eng.* 1–13. 10.1080/10255842.2023.2254881 (2023). 10.1080/10255842.2023.2254881 37713212

[39] Batagov, A. et al. Generalized metabolic flux analysis framework provides mechanism-based predictions of ophthalmic complications in type 2 diabetes patients. *Health Inf. Sci. Syst.* **11**, 18 (2023). 37008895 10.1007/s13755-023-00218-x PMC10060506

[40] Benattia, B. Z., Houda Maicha, N. E., Kerrache, C. A., Rathee, G. & Calafate, C. T. Implementing a blockchain based system for healthcare applications using digital twin. In *Proc. 2023 11th International Conference on Intelligent Systems and Embedded Design (ISED).* 1–6 (IEEE, Dehradun, India, 2023). 10.1109/ISED59382.2023.10444595.

[41] Arabidarrehdor, G. et al. Mathematical modeling, in-human evaluation and analysis of volume kinetics and kidney function after burn injury and resuscitation. *TBME* **69**, 366–376 (2022). 10.1109/TBME.2021.3094515 34236959

[42] Cen, S., Gebregziabher, M., Moazami, S., Azevedo, C. & Pelletier, D. Toward precision medicine using a ‘digital twin’ approach: modeling the onset of disease-specific brain atrophy in individuals with multiple sclerosis. *Res. Sq.*10.21203/rs.3.rs-2833532/v1 (2023). 37770560 10.1038/s41598-023-43618-5 PMC10539386

[43] Shu, H. et al. Twin-S: a digital twin for skull base surgery. *Int. J. Comput. Assist. Radio. Surg.* **18**, 1077–1084 (2023). 10.1007/s11548-023-02863-9 PMC11110948 37160583

[44] Chiaravalloti, A., Tomasi, C., Corsi, C. & Falanga, M. A digital twin approach for stroke risk assessment in atrial fibrillation patients. MetroXRAINE 17–21 10.1109/MetroXRAINE58569.2023.10405787 (2023). 10.1016/j.heliyon.2024.e39527 PMC11541462 39512324

[45] Surian, N. U. et al. A digital twin model incorporating generalized metabolic fluxes to identify and predict chronic kidney disease in type 2 diabetes mellitus. *NPJ Digital Med.* **7**, 140 (2024). 10.1038/s41746-024-01108-6 PMC11126707 38789510

[46] Sizemore, N. et al. A digital twin of the infant microbiome to predict neurodevelopmental deficits. *Sci. Adv.* **10**, eadj0400 (2024). 38598636 10.1126/sciadv.adj0400 PMC11006218

[47] Zhang, Y. et al. A framework towards digital twins for type 2 diabetes. *Front. Digit. Health* **6**, 1336050 (2024). 38343907 10.3389/fdgth.2024.1336050 PMC10853398

[48] Hussain, I., Hossain, M. A. & Park, S. J. A healthcare digital twin for diagnosis of stroke. In *Proc. 2021 IEEE International Conference on Biomedical Engineering, Computer and Information Technology for Health (BECITHCON).* 18–21 (IEEE, Dhaka, Bangladesh, 2021). 10.1109/BECITHCON54710.2021.9893641.

[49] Kolokotroni, E. et al. A multidisciplinary hyper-modeling scheme in personalized in silico oncology: coupling cell kinetics with metabolism, signaling networks, and biomechanics as plug-in component models of a cancer digital twin. *J. Pers. Med.* **14**, 475 (2024). 38793058 10.3390/jpm14050475 PMC11122096

[50] Lee, J. D. et al. A probabilistic neural twin for treatment planning in peripheral pulmonary artery stenosis. *Int. J. Numer. Method Biomed. Eng.* **40**, e3820 (2024). 38544354 10.1002/cnm.3820 PMC11131421

[51] Goodwin, G. C. et al. A systematic stochastic design strategy achieving an optimal tradeoff between peak BGL and probability of hypoglycaemic events for individuals having type 1 diabetes mellitus. *Biomed. Signal. Process Control* **57**, 101813 (2020).

[52] Xing, L., Liu, W., Liu, X. & Li, X. An enhanced vision transformer model in digital twins powered internet of medical things for pneumonia diagnosis. *JSAC* **41**, 3677–3689 (2023).

[53] Serra, D. et al. Assessment of risk for ventricular tachycardia based on extensive electrophysiology simulations. *Annu. Int. Conf. IEEE Eng. Med. Biol. Soc.* **2023**, 1–4 (2023). 38083190 10.1109/EMBC40787.2023.10340169

[54] Guy, A., Coulombe, M., Labelle, H., Barchi, S. & Aubin, C. Automated design of nighttime braces for adolescent idiopathic scoliosis with global shape optimization using a patient-specific finite element model. *Sci. Rep.* **14**, 3300 (2024). 38332053 10.1038/s41598-024-53586-z PMC10853218

[55] O’Hara, R. P. et al. Cardiac MRI oversampling in heart digital twins improves preprocedure ventricular tachycardia identification in postinfarction patients. *JACC Clin. Electrophysiol.*10.1016/j.jacep.2024.04.032 (2024). 38934970 10.1016/j.jacep.2024.04.032

[56] Aubert, K. et al. Development of digital twins to optimize trauma surgery and postoperative management. A case study focusing on tibial plateau fracture. *Front. Bioeng. Biotechnol.* **9**, 722275 (2021). 34692655 10.3389/fbioe.2021.722275 PMC8529153

[57] Silfvergren, O. et al. Digital twin predicting diet response before and after long-term fasting. *PLoS Comput. Biol.* **18**, e1010469 (2022). 36094958 10.1371/journal.pcbi.1010469 PMC9499255

[58] Tai, Y. et al. Digital-twin-enabled IoMT system for surgical simulation using rAC-GAN. *IEEE Internet Things J* **9**, 20918–20931 (2022).

[59] Zhang, Y. et al. DT-CTNet: a clinically interpretable diagnosis model for fetal distress. *Biomed. Signal. Process Control* **86**, 105190 (2023).

[60] Ložek, M. et al. How to assess and treat right ventricular electromechanical dyssynchrony in post-repair tetralogy of Fallot: insights from imaging, invasive studies, and computational modelling. *Europace* **26**, 1–9 (2024). 10.1093/europace/euae024 PMC10838147 38266248

[61] Rodero, C. et al. Impact of anatomical reverse remodelling in the design of optimal quadripolar pacing leads: a computational study. *Comput. Biol. Med.* **140**, 105073 (2022). 34852973 10.1016/j.compbiomed.2021.105073 PMC8752960

[62] Moztarzadeh, O. et al. Metaverse and medical diagnosis: a blockchain-based digital twinning approach based on MobileNetV2 algorithm for cervical vertebral maturation. *Diagnostics* **13**, 1485 (2023). 37189587 10.3390/diagnostics13081485 PMC10137959

[63] Checcucci, E. et al. Metaverse surgical planning with three-dimensional virtual models for minimally invasive partial nephrectomy. *Eur. Urol.* **85**, 320–325 (2024). 37673751 10.1016/j.eururo.2023.07.015

[64] Maleki, A. et al. Moving forward through the in silico modeling of multiple sclerosis: treatment layer implementation and validation. *Comput. Struct. Biotechnol. J* **21**, 3081–3090 (2023). 37266405 10.1016/j.csbj.2023.05.020 PMC10230825

[65] Wu, C. et al. MRI-based digital models forecast patient-specific treatment responses to neoadjuvant chemotherapy in triple-negative breast cancer. *Cancer Res.* **82**, 3394–3404 (2022). 35914239 10.1158/0008-5472.CAN-22-1329 PMC9481712

[66] Azzolin, L. et al. Personalized ablation vs. conventional ablation strategies to terminate atrial fibrillation and prevent recurrence. *Europace* **25**, 211–222 (2023). 35943361 10.1093/europace/euac116 PMC9907752

[67] Golse, N. et al. Predicting the risk of post-hepatectomy portal hypertension using a digital twin: a clinical proof of concept. *J. Hepatol.* **74**, 661–669 (2021). 33212089 10.1016/j.jhep.2020.10.036

[68] Chaudhuri, A. et al. Predictive digital twin for optimizing patient-specific radiotherapy regimens under uncertainty in high-grade gliomas. *Front. Artif. Intell.* **6**, 1222612 (2023). 37886348 10.3389/frai.2023.1222612 PMC10598726

[69] Geitner, C. M. et al. Pressure- and time-dependent alveolar recruitment/derecruitment in a spatially resolved patient-specific computational model for injured human lungs. *Int. J. Numer. Method Biomed. Eng.* **40**, e3787 (2024). 38037251 10.1002/cnm.3787

[70] Wan, Z., Dong, Y., Yu, Z., Lv, H. & Lv, Z. Semi-supervised support vector machine for digital twins based brain image fusion. *Front. Neurosci.* **15**, 705323 (2021). 34305523 10.3389/fnins.2021.705323 PMC8298822

[71] Servin, F. et al. Simulation of image-guided microwave ablation therapy using a digital twin computational model. *IEEE Open J. Eng. Med. Biol.* **5**, 107–124 (2024). 38445239 10.1109/OJEMB.2023.3345733 PMC10914207

[72] Koopsen, T. et al. Virtual pacing of a patient’s digital twin to predict left ventricular reverse remodelling after cardiac resynchronization therapy. *Europace* **26**, 1–8 (2023). 10.1093/europace/euae009 PMC10825733 38288616

[73] Zhou, C. et al. Virtual patients for mechanical ventilation in the intensive care unit. *Comput. Methods Prog. Biomed.* **199**, 105912 (2021). 10.1016/j.cmpb.2020.105912 33360683

[74] Langton, C. M., Grimm, A., Lloyd, D. G. & Frossard, L. A. A 3D-printed phantom twin and multi-transducer holder for dynamic anatomical ultrasonography of the lower limb. *J. 3D Print. Med.* **7**, 3DP009 (2023).

[75] Ahmadian, H. et al. A digital twin for simulating the vertebroplasty procedure and its impact on mechanical stability of vertebra in cancer patients. *Int. J. Numer. Method Biomed. Eng.* **38**, e3600 (2022). 35347880 10.1002/cnm.3600 PMC9287026

[76] Gillette, K. et al. A Framework for the generation of digital twins of cardiac electrophysiology from clinical 12-leads ECGs. *Med. Image Anal.* **71**, 102080 (2021). 33975097 10.1016/j.media.2021.102080

[77] Podéus, H. et al. A physiologically-based digital twin for alcohol consumption—predicting real-life drinking responses and long-term plasma PEth. *NPJ Digital Med.* **7**, 112 (2024). 10.1038/s41746-024-01089-6 PMC11068902 38702474

[78] Chakshu, N. K., Carson, J., Sazonov, I. & Nithiarasu, P. A semi-active human digital twin model for detecting severity of carotid stenoses from head vibration-A coupled computational mechanics and computer vision method. Int. J. Numer. *Method Biomed. Eng.* **35**, e3180 (2019). 10.1002/cnm.3180 PMC6593817 30648344

[79] Hassanzadeh, H., Boyle, J. & Khanna, S. A step towards building health digital twins: patient phenotype representation for health outcome prediction. *Stud. Health Technol. Inf.* **310**, 1011–1015 (2024). 10.3233/SHTI231117 38269967

[80] Hirschvogel, M., Jagschies, L., Maier, A., Wildhirt, S. M. & Gee, M. W. An in silico twin for epicardial augmentation of the failing heart. *Int. J. Numer. Method Biomed. Eng.* **35**, e3233 (2019). 31267697 10.1002/cnm.3233

[81] Jung, A., Gsell, M. A. F., Augustin, C. M. & Plank, G. An integrated workflow for building digital twins of cardiac electromechanics—a multi-fidelity approach for personalising active mechanics. *Mathematics* **10**, 823 (2022). 35295404 10.3390/math10050823 PMC7612499

[82] Männle, D. et al. Artificial intelligence directed development of a digital twin to measure soft tissue shift during head and neck surgery. *PLoS ONE* **18**, e0287081 (2023). 37556451 10.1371/journal.pone.0287081 PMC10411805

[83] Dubs, L. et al. Assessment of extracranial carotid artery disease using digital twins—a pilot study. *NeuroImage Clin.* **38**, 103435 (2023). 37245493 10.1016/j.nicl.2023.103435 PMC10238877

[84] Azzolin, L. et al. AugmentA: patient-specific augmented atrial model generation tool. *Comput. Med. Imaging Graph* **108**, 102265 (2023). 37392493 10.1016/j.compmedimag.2023.102265

[85] Chakshu, N. K., Carson, J. M., Sazonov, I. & Nithiarasu, P. Automating fractional flow reserve (FFR) calculation from CT scans: a rapid workflow using unsupervised learning and computational fluid dynamics. *Int. J. Numer. Method Biomed. Eng.* **38**, e3559 (2022). 34865317 10.1002/cnm.3559 PMC11475381

[86] Ishida, H. et al. Beyond the manual touch: situational-aware force control for increased safety in robot-assisted skullbase surgery. *Int. J. Comput. Assist. Radio. Surg.*10.1007/s11548-024-03168-1 (2024). 10.1007/s11548-024-03168-1 38816649

[87] Rouhollahi, A. et al. CardioVision: a fully automated deep learning package for medical image segmentation and reconstruction generating digital twins for patients with aortic stenosis. *Comput. Med. Imaging Graph* **109**, 102289 (2023). 37633032 10.1016/j.compmedimag.2023.102289 PMC10599298

[88] Strocchi, M. et al. Cell to whole organ global sensitivity analysis on a four-chamber heart electromechanics model using Gaussian processes emulators. *PLoS Comput. Biol.* **19**, e1011257 (2023). 37363928 10.1371/journal.pcbi.1011257 PMC10328347

[89] Jeising, S. et al. Combined use of 3D printing and mixed reality technology for neurosurgical training: getting ready for brain surgery. *Neurosurg. Focus* **56**, E12 (2024). 38163360 10.3171/2023.10.FOCUS23611

[90] Cho, R. Y. et al. Comparative analysis of three facial scanners for creating digital twins by focusing on the difference in scanning method. *Bioengineering* **10**, 545 (2023). 37237615 10.3390/bioengineering10050545 PMC10215089

[91] Sarantides, P. et al. Computational study of abdominal aortic aneurysm walls accounting for patient-specific non-uniform intraluminal thrombus thickness and distinct material models: a pre- and post-rupture case. *Bioengineering* **11**, 144 (2024). 38391630 10.3390/bioengineering11020144 PMC10886172

[92] Demir, O., Uslan, I., Buyuk, M. & Salamci, M. U. Development and validation of a digital twin of the human lower jaw under impact loading by using non-linear finite element analyses. *J. Mech. Behav. Biomed. Mater* **148**, 106207 (2023). 37922761 10.1016/j.jmbbm.2023.106207

[93] Yang, Q. et al. Development of digital fetal heart models with virtual ultrasound function based on cardiovascular casting and computed tomography scan. *Bioengineering* **9**, 524 (2022). 36290492 10.3390/bioengineering9100524 PMC9598759

[94] Salvador, M. et al. Digital twinning of cardiac electrophysiology for congenital heart disease. *J. R. Soc. Interface* **21**, 20230729 (2024). 38835246 10.1098/rsif.2023.0729 PMC11285762

[95] Grandits, T., Verhülsdonk, J., Haase, G., Effland, A. & Pezzuto, S. Digital twinning of cardiac electrophysiology models from the surface ECG: a geodesic backpropagation approach. *TBME* **71**, 1281–1288 (2024). 10.1109/TBME.2023.3331876 38048238

[96] Camps, J. et al. Digital twinning of the human ventricular activation sequence to clinical 12-lead ECGs and magnetic resonance imaging using realistic Purkinje networks for in silico clinical trials. *Med. Image Anal.* **94**, 103108 (2024). 38447244 10.1016/j.media.2024.103108

[97] Hernigou, P. et al. Digital twins, artificial intelligence, and machine learning technology to identify a real personalized motion axis of the tibiotalar joint for robotics in total ankle arthroplasty. *Int. Orthop.* **45**, 2209–2217 (2021). 34351462 10.1007/s00264-021-05175-2

[98] Lee, J. H. et al. Effectiveness of creating digital twins with different digital dentition models and cone-beam computed tomography. *Sci. Rep.* **13**, 10603 (2023). 37391453 10.1038/s41598-023-37774-x PMC10313775

[99] Straughan, R., Kadry, K., Parikh, S. A., Edelman, E. R. & Nezami, F. R. Fully automated construction of three-dimensional finite element simulations from Optical Coherence Tomography. *Comput. Biol. Med.* **165**, 107341 (2023). 37611423 10.1016/j.compbiomed.2023.107341 PMC10528179

[100] Barbiero, P., Torné, R. V. & Lió, P. Graph representation forecasting of patient’s medical conditions: toward a digital twin. *Front. Genet.* **12**, 652907 (2021). 34603366 10.3389/fgene.2021.652907 PMC8481902

[101] Xing, X. et al. HDL: hybrid deep learning for the synthesis of myocardial velocity maps in digital twins for cardiac analysis. *J. Biomed. Health Inform.* **27**, 5134–5142 (2023). 10.1109/JBHI.2022.3158897 35290192

[102] Grob, M., Seisl, P., Rappelsberger, A. & Adlassnig, K. P. Health digital twins with clinical decision support. *Stud. Health Technol. Inf.* **305**, 151–152 (2023). 10.3233/SHTI230448 37386982

[103] Michaux, P., Gaume, B., Cong, Y. & Quéméner, O. Human body numerical simulation: an accurate model for a thigh subjected to a cold treatment. *Comput. Biol. Med.* **168**, 107689 (2024). 37984207 10.1016/j.compbiomed.2023.107689

[104] Ibrahim, M. T., Majumder, A., Gopi, M., Sayadi, L. R. & Vyas, R. M. Illuminating precise stencils on surgical sites using projection-based augmented reality. *Smart Health* **32**, 100476 (2024).

[105] Lu, W. et al. Imitating and exploring the human brain’s resting and task-performing states via brain computing: scaling and architecture. *Natl. Sci. Rev.* **11**, nwae080 (2024). 38803564 10.1093/nsr/nwae080 PMC11129584

[106] Förster, K. M. et al. In silico numerical simulation of ventilator settings during high-frequency ventilation in preterm infants. *Pediatr. Pulmonol.* **56**, 3839–3846 (2021). 34432956 10.1002/ppul.25626

[107] Boillet, A., Messonnier, L. A. & Cohen, C. Individualized physiology-based digital twin model for sports performance prediction: a reinterpretation of the Margaria-Morton model. *Sci. Rep.* **14**, 5470 (2024). 38443504 10.1038/s41598-024-56042-0 PMC10915161

[108] Camps, J. et al. Inference of number and location of purkinje root nodes and ventricular conduction properties from clinical 12-lead ECGs for cardiac digital twinning. *CinC* **498**, 1–4 (2022).

[109] Bjelland, Ø., Pedersen, M. D., Steinert, M. & Bye, R. T. Intraoperative data-based haptic feedback for arthroscopic partial meniscectomy punch simulation. *IEEE Access* **10**, 107269–107282 (2022).

[110] Fu, W. et al. IPhantom: a framework for automated creation of individualized computational phantoms and its application to CT organ dosimetry. *J. BHI* **25**, 3061–3072 (2021). 10.1109/JBHI.2021.3063080 PMC8502243 33651703

[111] Lachinov, D., Chakravarty, A., Grechenig, C., Schmidt-Erfurth, U. & Bogunovic, H. Learning spatio-temporal model of disease progression with NeuralODEs from longitudinal volumetric data. *IEEE Trans. Med. Imaging* **43**, 1165–1179 (2024). 37934647 10.1109/TMI.2023.3330576

[112] Avanzato, R., Beritelli, F., Lombardo, A. & Ricci, C. Lung-DT: an AI-powered digital twin framework for thoracic health monitoring and diagnosis. *Sensors* **24**, 958 (2024). 38339678 10.3390/s24030958 PMC10857717

[113] Kirkels, F. P. et al. Monitoring of myocardial involvement in early arrhythmogenic right ventricular cardiomyopathy across the age spectrum. *JACC.* **82**, 785–797 (2023). 37612010 10.1016/j.jacc.2023.05.065

[114] Lakshminarayanan, K. et al. Motor imagery performance through embodied digital twins in a virtual reality-enabled brain-computer interface environment. *J. Vis. Exp.*10.3791/66859 (2024). 38801273 10.3791/66859

[115] Comte, N. et al. Multi-modal data correspondence for the 4D analysis of the spine with adolescent idiopathic scoliosis. *Bioengineering* **10**, 874 (2023). 37508901 10.3390/bioengineering10070874 PMC10376049

[116] Koopsen, T. et al. Parameter subset reduction for imaging-based digital twin generation of patients with left ventricular mechanical discoordination. *Biomed. Eng. Online* **23**, 46 (2024). 38741182 10.1186/s12938-024-01232-0 PMC11089736

[117] Geissler, F. et al. Personalized computed tomography—automated estimation of height and weight of a simulated digital twin using a 3D camera and artificial intelligence. *RoFo* **193**, 437–445 (2021). 33142337 10.1055/a-1253-8558

[118] Caljé-van der Klei, T. et al. Pulmonary response prediction through personalized basis functions in a virtual patient model. *Comput. Methods Prog. Biomed.* **244**, 107988 (2024). 10.1016/j.cmpb.2023.107988 38171168

[119] Cappon, G., Vettoretti, M., Sparacino, G., Del Favero, S. & Facchinetti, A. ReplayBG: a digital twin-based methodology to identify a personalized model from type 1 diabetes data and simulate glucose concentrations to assess alternative therapies. *TBME* **70**, 3227–3238 (2023). 10.1109/TBME.2023.3286856 37368794

[120] Cho, S. W. et al. Sagittal relationship between the maxillary central incisors and the forehead in digital twins of Korean adult females. *J. Pers. Med.* **11**, 203 (2021). 33805617 10.3390/jpm11030203 PMC8001265

[121] Wachter, A., Kost, J. & Nahm, W. Simulation-based estimation of the number of cameras required for 3D reconstruction in a narrow-baseline multi-camera setup. *J. Imaging* **7**, 87 (2021). 34460683 10.3390/jimaging7050087 PMC8321353

[122] Hernigou, P., Safar, A., Hernigou, J. & Ferre, B. Subtalar axis determined by combining digital twins and artificial intelligence: influence of the orientation of this axis for hindfoot compensation of varus and valgus knees. *Int. Orthop.* **46**, 999–1007 (2022). 35138455 10.1007/s00264-022-05311-6

[123] Ahmadian, H. et al. Toward an artificial intelligence-assisted framework for reconstructing the digital twin of vertebra and predicting its fracture response. *Int. J. Numer. Method Biomed. Eng.* **38**, e3601 (2022). 35403831 10.1002/cnm.3601 PMC9285948

[124] Li, L. et al. Toward enabling cardiac digital twins of myocardial infarction using deep computational models for inverse inference. *IEEE Trans. Med. Imaging* **43**, 2466–2478 (2024). 38373128 10.1109/TMI.2024.3367409 PMC7616288

[125] Doste, R. et al. Training machine learning models with synthetic data improves the prediction of ventricular origin in outflow tract ventricular arrhythmias. *Front. Physiol.* **13**, 909372 (2022). 36035489 10.3389/fphys.2022.909372 PMC9412034

[126] van Osta, N. et al. Uncertainty quantification of regional cardiac tissue properties in arrhythmogenic cardiomyopathy using adaptive multiple importance sampling. *Front. Physiol.* **12**, 738926 (2021). 34658923 10.3389/fphys.2021.738926 PMC8514656

[127] Ang, C. Y. S. et al. Virtual patient framework for the testing of mechanical ventilation airway pressure and flow settings protocol. *Comput. Methods Prog. Biomed.* **226**, 107146 (2022). 10.1016/j.cmpb.2022.107146 36191352

[128] Salvador, M. et al. Whole-heart electromechanical simulations using latent neural ordinary differential equations. *NPJ Digital Med.* **7**, 90 (2024). 10.1038/s41746-024-01084-x PMC11009296 38605089

[129] Zhao, J. et al. A liver digital twin for in silico testing of cellular and inter-cellular mechanisms in regeneration after drug-induced damage. *iScience* **27**, 108077 (2024). 38371522 10.1016/j.isci.2023.108077 PMC10869925

[130] Herrgårdh, T. et al. A multi-scale digital twin for adiposity-driven insulin resistance in humans: diet and drug effects. *Diabetol. Metab. Syndr.* **15**, 250 (2023). 38044443 10.1186/s13098-023-01223-6 PMC10694923

[131] Yang, P. C. et al. A multiscale predictive digital twin for neurocardiac modulation. *J. Physiol.* **601**, 3789–3812 (2023). 37528537 10.1113/JP284391 PMC10528740

[132] Maksymenko, K., Clarke, A. K., Mendez Guerra, I., Deslauriers-Gauthier, S. & Farina, D. A myoelectric digital twin for fast and realistic modelling in deep learning. *Nat. Commun.* **14**, 1600 (2023). 36959193 10.1038/s41467-023-37238-w PMC10036636

[133] Cimpeanu, R., Castrejón-Pita, A. A., Lim, L. N., Vatish, M. & Georgiou, E. X. A new flow-based design for double-lumen needles. *J. Biomech.* **160**, 111832 (2023). 37837837 10.1016/j.jbiomech.2023.111832

[134] Rovati, L. et al. Development and usability testing of a patient digital twin for critical care education: a mixed methods study. *Front. Med.* **10**, 1336897 (2023). 10.3389/fmed.2023.1336897 PMC10808677 38274456

[135] Hoehme, S. et al. Digital twin demonstrates significance of biomechanical growth control in liver regeneration after partial hepatectomy. *iScience* **26**, 105714 (2023). 36691615 10.1016/j.isci.2022.105714 PMC9860368

[136] Migliaccio, G. et al. Exploring cell migration mechanisms in cancer: from wound healing assays to cellular automata models. *Cancers* **15**, 5284 (2023). 37958456 10.3390/cancers15215284 PMC10647277

[137] Viola, F., Del Corso, G., De Paulis, R. & Verzicco, R. GPU accelerated digital twins of the human heart open new routes for cardiovascular research. *Sci. Rep.* **13**, 8230 (2023). 37217483 10.1038/s41598-023-34098-8 PMC10203142

[138] Terpsma, R. et al. Head impact modeling to support a rotational combat helmet drop test. *Mil. Med.* **188**, e745–e752 (2023). 34508268 10.1093/milmed/usab374

[139] Dichamp, J. et al. In vitro to in vivo acetaminophen hepatotoxicity extrapolation using classical schemes, pharmacodynamic models and a multiscale spatial-temporal liver twin. *Front. Bioeng. Biotechnol.* **11**, 1049564 (2023). 36815881 10.3389/fbioe.2023.1049564 PMC9932319

[140] Calka, M. et al. Machine-learning based model order reduction of a biomechanical model of the human tongue. *Comput. Methods Prog. Biomed.* **198**, 105786 (2021). 10.1016/j.cmpb.2020.105786 33059060

[141] Hoang, D., Kuang, B., Liang, G., Wang, Z. & Yoon, S. Modulation of nutrient precursors for controlling metabolic inhibitors by genome-scale flux balance analysis. *Biotechnol. Prog.* **39**, e3313 (2023). 36367527 10.1002/btpr.3313

[142] Mazumder, O., Roy, D., Bhattacharya, S., Sinha, A. & Pal, A. Synthetic PPG generation from haemodynamic model with baroreflex autoregulation: a digital twin of cardiovascular system. *EMBC* **5024**, 5029 (2019). 10.1109/EMBC.2019.8856691 31946988

[143] Michaud, F., Luaces, A., Mouzo, F. & Cuadrado, J. Use of patellofemoral digital twins for patellar tracking and treatment prediction: comparison of 3D models and contact detection algorithms. *Front. Bioeng. Biotechnol.* **12**, 1347720 (2024). 38481569 10.3389/fbioe.2024.1347720 PMC10935559

[144] Venkatapurapu, S. P. et al. A computational platform integrating a mechanistic model of Crohn’s disease for predicting temporal progression of mucosal damage and healing. *Adv. Ther.* **39**, 3225–3247 (2022). 35581423 10.1007/s12325-022-02144-y PMC9239932

[145] Grieb, N. et al. A digital twin model for evidence-based clinical decision support in multiple myeloma treatment. *Front. Digit. Health* **5**, 1324453 (2023). 38173909 10.3389/fdgth.2023.1324453 PMC10761485

[146] Reimann, A. M. et al. AML consolidation therapy: timing matters. *J. Cancer Res. Clin. Oncol* **149**, 13811–13821 (2023). 37535164 10.1007/s00432-023-05115-0 PMC10590325

[147] Lin, T. Y. et al. Assessing overdiagnosis of fecal immunological test screening for colorectal cancer with a digital twin approach. *NPJ Digital Med.* **6**, 24 (2023). 10.1038/s41746-023-00763-5 PMC9918445 36765093

[148] Chandra, S., Prakash, P. K. S., Samanta, S. & Chilukuri, S. ClinicalGAN: powering patient monitoring in clinical trials with patient digital twins. *Sci. Rep.* **14**, 12236 (2024). 38806536 10.1038/s41598-024-62567-1 PMC11133486

[149] Li, G., Chen, Y. B. & Peachey, J. Construction of a digital twin of chronic graft vs. host disease patients with standard of care. *BMT*10.1038/s41409-024-02324-0 (2024). 10.1038/s41409-024-02324-0 PMC11368802 38898224

[150] Young, G. et al. Design and in silico evaluation of an exercise decision support system using digital twin models. *J. Diab. Sci. Technol.* **18**, 324–334 (2024). 10.1177/19322968231223217 PMC10973845 38390855

[151] Joslyn, L. R., Huang, W., Miles, D., Hosseini, I. & Ramanujan, S. Digital twins elucidate critical role of T(scm) in clinical persistence of TCR-engineered cell therapy. *NPJ Syst. Biol. Appl.* **10**, 11 (2024). 38278838 10.1038/s41540-024-00335-7 PMC10817974

[152] Franz, S. et al. Impact of heterotopic ossification on functional recovery in acute spinal cord injury. *Front. Cell Neurosci.* **16**, 842090 (2022). 35221928 10.3389/fncel.2022.842090 PMC8864137

[153] Coleman, J. A. et al. Mechanisms of ischaemia-induced arrhythmias in hypertrophic cardiomyopathy: a large-scale computational study. *Cardiovasc. Res.* **120**, 914–926 (2024). 38646743 10.1093/cvr/cvae086 PMC11218689

[154] Moore, J. H. et al. SynTwin: a graph-based approach for predicting clinical outcomes using digital twins derived from synthetic patients. *Pac. Symp. Biocomput.* **29**, 96–107 (2024). 38160272 PMC10827004

[155] Susilo, M. E. et al. Systems-based digital twins to help characterize clinical dose–response and propose predictive biomarkers in a phase I study of bispecific antibody, mosunetuzumab, in NHL. *CTS* **16**, 1134–1148 (2023). 36908269 10.1111/cts.13501 PMC10339700

[156] Yang, P. C. et al. Toward digital twin technology for precision pharmacology. *JACC Clin. Electrophysiol.* **10**, 359–364 (2024). 38069976 10.1016/j.jacep.2023.10.024 PMC12049087

[157] Qi, T. & Cao, Y. Virtual clinical trials: a tool for predicting patients who may benefit from treatment beyond progression with pembrolizumab in non-small cell lung cancer. *CPT Pharmacomet. Syst. Pharm.* **12**, 236–249 (2023). 10.1002/psp4.12896 PMC9931430 36547213

[158] Trayanova, N. A. & Prakosa, A. Up digital and personal: how heart digital twins can transform heart patient care. *Heart Rhythm* **21**, 89–99 (2024). 37871809 10.1016/j.hrthm.2023.10.019 PMC10872898

[159] ASME. V&V 10—Verification and Validation in Computational Solid Mechanics (American Society of Mechanical Engineering, 2006).

[160] ASME. V&V 20—Verification and Validation in Computational Fluid Dynamics and Heat Transfer (American Society of Mechanical Engineering, 2009).

[161] ASME. V&V 40—Assessing Credibility of Computational Modeling through Verification and Validation: Application to Medical Devices (ASME, 2018).

[162] Galappaththige, S., Gray, R. A., Costa, C. M., Niederer, S. & Pathmanathan, P. Credibility assessment of patient-specific computational modeling using patient-specific cardiac modeling as an exemplar. *PLoS Comput. Biol.* **18**, e1010541 (2022). 36215228 10.1371/journal.pcbi.1010541 PMC9550052

[163] Santiago, A. et al. Design and execution of a verification, validation, and uncertainty quantification plan for a numerical model of left ventricular flow after LVAD implantation. *PLoS Comput. Biol.* **18**, e1010141 (2022). 35696442 10.1371/journal.pcbi.1010141 PMC9232142

[164] Nagaraja, S. et al. Verification, validation, and uncertainty quantification of spinal rod computational models under three-point bending. *J. Verif. Valid. Uncert.* **5**, 011002 (2020).

[165] Covidence Systematic Review Software. Veritas Health Innovation.

[166] Chase, J. G., Desaive, T. & Preiser, J.-C. Virtual patients and virtual cohorts: a new way to think about the design and implementation of personalized ICU treatments. In *Annual Update in Intensive Care and Emergency Medicine 2016* (ed. Vincent, J.-L.) 435–448 (Springer International Publishing, 2016).

